# Baseline Wearable Turning-Derived Signature and Dopamine Transporter-Derived Measures in Relation to Subsequent Motor Progression in Parkinson’s Disease: An Exploratory Longitudinal Study

**DOI:** 10.3390/s26206610

**Published:** 2026-10-19

**Authors:** Hwayoung Park, Changhong Youm, Bohyun Kim, Juseon Hwang, Minsoo Kim, Sang-Myung Cheon, Young Jin Jeong

**Affiliations:** 1 Biomechanics Laboratory, Dong-A University, Saha-gu, Busan 49315, Republic of Korea; app00113@donga.ac.kr (H.P.); 2177638@donga.ac.kr (B.K.); 2577561@donga.ac.kr (J.H.); 2677610@donga.ac.kr (M.K.); 2 Department of Health Sciences, The Graduate School, Dong-A University, Saha-gu, Busan 49315, Republic of Korea; 3 Department of Neurology, School of Medicine, Dong-A University, Seo-gu, Busan 49201, Republic of Korea; smcheon@dau.ac.kr; 4 Department of Nuclear Medicine, Dong-A University Hospital, College of Medicine, Dong-A University, Seo-gu, Busan 49201, Republic of Korea; nmjeong@dau.ac.kr

**Keywords:** Parkinson’s disease, wearable sensors, inertial measurement units, turning-derived signature, gait analysis, dopamine transporter imaging, motor progression, ^18^F-FP-CIT PET, digital biomarkers, longitudinal assessment

## Abstract

**Highlights:**

**What are the main findings?**

- Baseline wearable turning-derived signature showed higher internally cross-validated discrimination of subsequent motor progression than the examined baseline DAT burden and asymmetry measures.
- A risk-aligned turning-derived signature was positively associated with subsequent worsening in UPDRS Part III and distinguished exploratory groups with different motor progression profiles.

**What are the implications of the main findings?**

- Wearable turning-derived signature may provide complementary functional information to baseline DAT imaging when characterizing motor progression in Parkinson’s disease.
- Independent validation in larger prospective cohorts is needed before the turning-derived signature can be considered for clinical prognostic use.

**Abstract:**

Parkinson’s disease (PD) shows heterogeneity in motor progression, complicating prognostic assessment. Dopamine transporter (DAT) imaging provides an in vivo measure of nigrostriatal dopaminergic integrity, whereas wearable gait assessment quantifies motor performance. However, the prognostic value of baseline (BL) DAT-derived measures and wearable gait characteristics remains incompletely understood. We investigated whether BL DAT-derived measures and a limb-focused wearable turning movement-dynamics signature (turning-derived signature) were associated with subsequent motor progression in PD. Twenty-three individuals with PD underwent BL PET imaging, wearable gait assessment, and clinical follow-up. Motor progression was defined using a cohort-specific exploratory threshold of ΔUnified Parkinson’s Disease Rating Scale (UPDRS) Part III ≥ 1.5 points, derived within this cohort using Hoehn and Yahr (H&Y)-stage worsening as the clinical anchor. Twelve participants were classified as motor progressors and 11 as non-progressors. The turning signature yielded an AUC of 0.750, while DAT-model AUCs ranged from 0.462 to 0.500. The clinical model yielded an AUC of 0.962; adding the turning signature yielded an AUC of 0.871. These findings suggest that the BL turning-derived signature contains an exploratory prognostic signal but does not establish incremental value beyond clinical severity. The progression outcome was derived within this small cohort, and external validation is required.

## 1. Introduction

Parkinson’s disease (PD) is a progressive neurodegenerative disorder characterized by heterogeneous motor and non-motor symptoms and variable rates of disease progression [1,2]. Dopaminergic neuronal loss in the nigrostriatal pathway is a defining biological feature of PD, with the clinical course varying markedly across individuals [2]. Recent biological staging frameworks have emphasized the need for PD-related disorders to be conceptualized using both biological anchors, including α-synuclein pathology and dopaminergic dysfunction, and the degree of functional impairment caused by clinical signs and symptoms [3]. Hence, biomarkers that reflect underlying biological pathology while also indexing the degree of functional impairment are urgently needed [3,4].

Dopamine transporter (DAT) imaging is widely used as an in vivo marker of presynaptic dopaminergic dysfunction. Molecular imaging of the dopamine system detects striatal dopaminergic changes and has been incorporated into emerging biological frameworks for neuronal α-synuclein disease [3]. Quantitative DAT imaging is associated with motor severity and disease stage in PD [5]. For example, striatal DAT binding has been associated with the Hoehn and Yahr (H&Y) stage, Unified Parkinson’s Disease Rating Scale (UPDRS) scores, rigidity, and axial scores in early PD cohorts [6]. Earlier ^18^F-FP-CIT positron emission tomography (PET) studies reported reduced uptake in striatal subregions and negative correlations between striatal uptake and motor UPDRS scores [5]. However, the extent to which baseline (BL) DAT imaging predicts subsequent functional motor decline is not established sufficiently. DAT imaging provides an in vivo snapshot of dopaminergic pathology [7], but prior prognostic studies have reported mixed prognostic findings, indicating that reduced striatal DAT binding is linked to the late onset of freezing of gait, whereas associations with falls, motor fluctuations, dyskinesia, and progression to H&Y stage 3 have not been consistently observed [7,8]. These findings suggest that BL dopaminergic uptake may not fully capture subsequent functional motor progression [8]. This may involve broader network-level and task-specific control demands beyond regional dopaminergic uptake [9].

This limitation is particularly relevant for gait and axial motor features. Gait abnormalities—including reduced gait speed, shortened step length, increased axial rigidity, impaired rhythmicity, freezing of gait, and progressive balance and postural control deficits—are significant motor symptoms of PD [9]. These symptoms strongly affect mobility, fall risk, independence, and quality of life [9]. Because gait and postural control involve distributed motor control systems, including basal ganglia, brainstem–spinal, cerebellar, and cortical networks, wearable sensor-derived gait features may reflect functional motor-network integrity [10,11]. Wearable sensors capture subtle spatiotemporal and segmental characteristics of gait and balance in laboratory, clinical, and potentially home-based settings for quantifying motor function in PD. However, because these data are often high-dimensional and noisy, clinically meaningful interpretation requires appropriate feature selection, dimensionality reduction, and validation against established clinical outcomes [12]. Therefore, recent digital gait studies examined whether wearable sensor-derived gait data are associated with UPDRS scores, disease severity stage, and longitudinal motor progression [13,14]. Raschka et al. reported that digital gait features related to UPDRS-derived progression outcomes. However, they also noted that cross-sectional digital gait features alone may have limited prognostic value and require careful validation [9].

Among gait tasks, turning may be particularly informative for PD progression because it requires whole-body reorientation, axial rotation, intersegmental coordination, dynamic balance, and task-specific motor adaptation [15]. Compared with steady-state straight walking, these demands may expose subtle motor control alterations related to dynamic instability, reduced compensatory capacity, and broader locomotor system dysfunction beyond nigrostriatal dopaminergic loss [16]. Accordingly, turning movement-dynamics features may serve as a task-specific marker of the consistency and stability of coordinated motor control during a challenging locomotor task [14,17]. Our previous sensor-based study using 360° turning tasks suggested that bilateral wearable sensor-derived features may capture clinically relevant asymmetry and severity-related gait disturbances in PD [12]. This functional information is conceptually distinct from DAT imaging, which reflects presynaptic dopaminergic integrity and provides an in vivo biological snapshot of dopaminergic pathology [3,7]. However, it remains unclear whether BL wearable sensor-derived turning movement dynamics offers prognostic information for subsequent motor progression beyond that captured by BL DAT uptake.

Therefore, this exploratory longitudinal study aimed to compare BL DAT uptake and wearable sensor-derived gait features for stratifying subsequent motor progression in PD. We hypothesized that task-specific wearable-derived turning movement-dynamics features would show a stronger association with subsequent ΔUPDRS Part III worsening and H&Y-anchored motor progression than BL DAT burden.

## 2. Materials and Methods

### 2.1. Study Design and Participants

This exploratory longitudinal study initially included 51 individuals with PD who underwent BL ^18^F-FP-CIT PET, clinical and wearable sensor-based gait assessments. Longitudinal assessments were conducted between November 2022 and December 2024, with all BL and final follow-up assessments conducted in the medication “ON” state, approximately 2–3 h after participants had taken their usual dopaminergic medication. This assessment window was used to standardize the medication context across visits and approximate the clinically defined “ON” state, rather than to confirm an individual pharmacological peak response. Participants were eligible for this analysis if BL ^18^F-FP-CIT PET data were available, demographic and clinical outcome data necessary for the longitudinal analysis were complete, and wearable sensor-derived gait features were present at both BL and final follow-up. The interval between the BL ^18^F-FP-CIT PET scan and the final follow-up assessment was available during the study period intended to capture approximately 2-year changes. The mean BL-to-final follow-up interval was 21.99 ± 1.93 months. The median interval was 21.75 months (interquartile range, 20.32–23.77 months), with participant-level intervals ranging from 19.25 to 24.87 months (Supplementary Materials Table S1).

**Figure 1:**
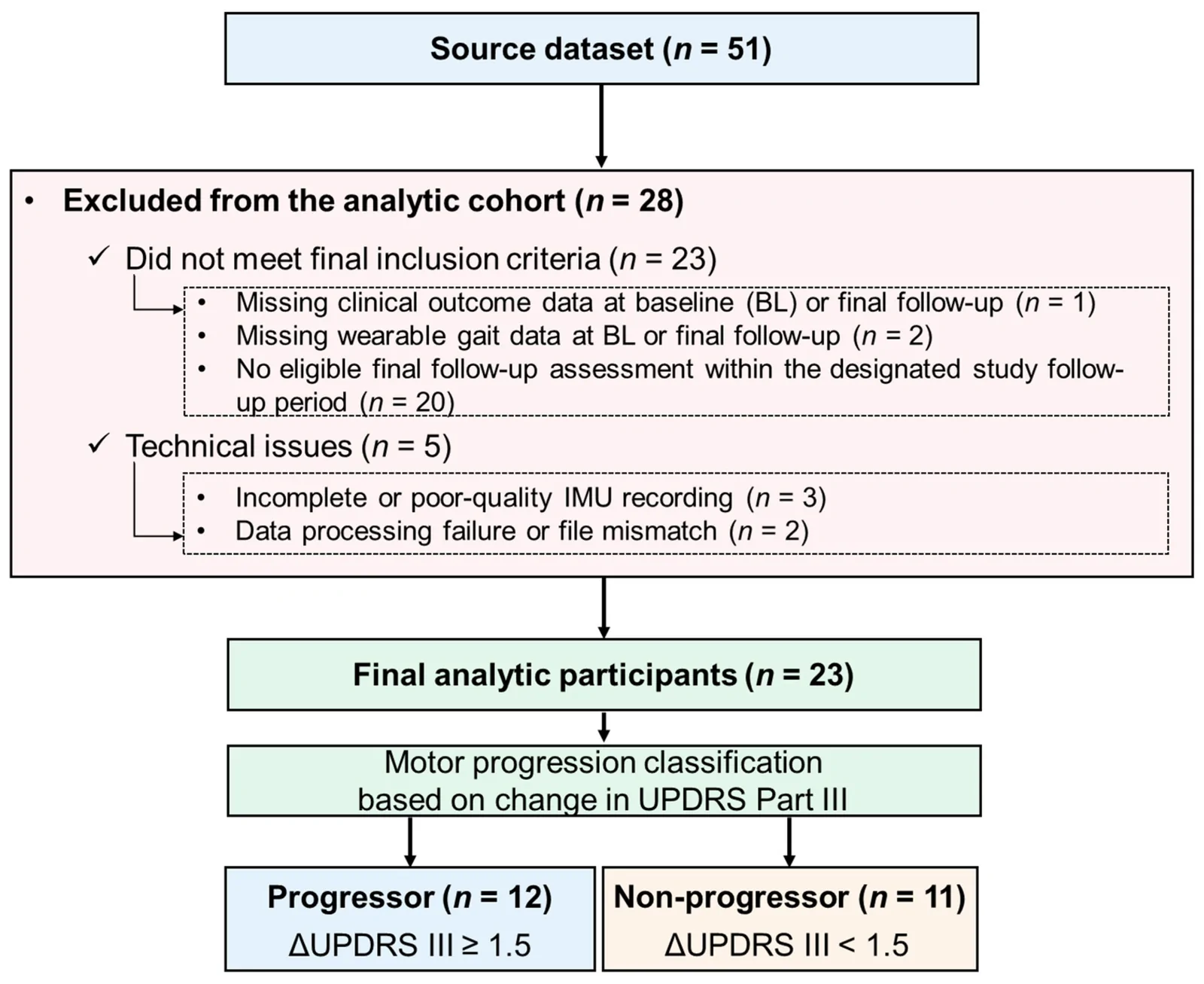
Participant flow diagram. Among the 23 participants in the final analysis, 12 were classified as progressors due to an increase of ≥1.5 points in UPDRS Part III from baseline to the final follow-up, whereas 11 were classified as non-progressors. IMU, inertial measurement unit; UPDRS, Unified Parkinson’s Disease Rating Scale.

Of the 51 participants in the source dataset, 28 were excluded from the analytic cohort. Of these, 23 did not meet the final inclusion criteria: one had missing clinical outcome data at BL or final follow-up, two had missing wearable gait data at BL or final follow-up, and 20 did not have an eligible final follow-up assessment within the designated study follow-up period. The remaining five participants were excluded because of technical issues, comprising incomplete or poor-quality inertial measurement unit (IMU) recordings (*n* = 3) and data-processing failure or file mismatch (*n* = 2). Based on these criteria, 23 individuals with PD (12 men and 11 women) were included in the final analytic sample. Although intermediate follow-up data were available, the primary prognostic analysis focused on whether BL DAT uptake and BL wearable sensor-derived gait features could stratify motor progression from BL to the final follow-up visit. The participant inclusion process is illustrated in Figure 1. Table 1 summarizes the demographic, clinical, and ^18^F-FP-CIT PET characteristics of the study participants used in the final analysis, stratified by motor progression status to characterize changes from BL to the final follow-up (Supplementary Materials Table S2). Follow-up duration was similar in study-defined motor progressors and non-progressors (21.82 ± 2.01 vs. 22.16 ± 1.92 months; Mann–Whitney U test, *p* = 0.689). Moreover, it was not associated with ΔUPDRS Part III (Spearman ρ = −0.046, *p* = 0.834), suggesting that longer follow-up intervals were not systematically associated with larger motor-score changes.

This study included individuals diagnosed with idiopathic PD according to the 2015 Movement Disorder Society (MDS) Clinical Diagnostic Criteria for Parkinson’s Disease by a clinician [18]. Inclusion criteria were age > 63 years and H&Y stage 1–3, indicating independent walking and mobility without assistive devices. Exclusion criteria included a history of cardiovascular, musculoskeletal, vestibular, or other neurological disorders; use of mobility-assistive devices; and pharmacotherapy-refractory dyskinesia. The Institutional Review Board of Dong-A University Hospital approved the study (IRB number: DAUHIRB–22-089). All participants received a full explanation of the study procedures and provided written informed consent. The study was registered in the Clinical Research Information Service of the Republic of Korea on 19 April 2024 (https://cris.nih.go.kr, registration number: KCT0009353).

### 2.2. Clinical Outcome

All participants underwent standardized physical assessments and clinician-administered evaluations of clinical characteristics. Cognitive function was evaluated using the validated Korean version of the Mini-Mental State Examination (K-MMSE) [19,20,21] and the Montreal Cognitive Assessment (MoCA-K) [22]. Motor severity was assessed using the UPDRS [23,24] and H&Y scale [25,26].

To complement the UPDRS Part III-based definition of motor progression, changes in the H&Y stage were used as a clinical anchor to derive a cohort-specific threshold. The resulting UPDRS Part III threshold was subsequently used to define the primary progressor classification. Between BL and the final follow-up, H&Y stage worsened in 9 of 23 participants (39.1%), remained unchanged in 13 (56.5%), and improved in one participant (4.3%). Specifically, seven participants transitioned from H&Y stage 1 to 2, two transitioned from stage 2 to 3, and one participant improved from stage 3 to 2 (Table 1).

### 2.3. Baseline ^18^F-FP-CIT Positron Emission Tomography Scan and DAT Uptake Quantification

BL ^18^F-FP-CIT PET images were acquired using a Biograph mCT flow PET/Computed Tomography (CT) scanner (Siemens Healthcare, Knoxville, TN, USA). A head fixation device minimized head motion during image acquisition. A low-dose brain CT scan was obtained for attenuation correction. PET images were acquired for 10 min, 3 h after intravenous injection of 185 MBq ^18^F-FP-CIT, from the skull vertex to the skull base. Three-dimensional PET images were reconstructed using an ordered-subsets expectation maximization algorithm with a 400 × 400 × 110 matrix and a voxel size of 2 × 2 × 2 mm. Two experienced nuclear medicine specialists reviewed all PET/CT images using a dedicated workstation equipped with customized software. PET images were co-registered with T1-weighted MRI using this software, and the Standardized Uptake Value Ratio (SUVR) was automatically calculated for specific brain regions (Striatum, caudate nucleus, and putamen) with the occipital cortex as the reference region.

The SUVR (standardized uptake value ratio) is a semiquantitative measure commonly used in PET imaging to assess regional tracer uptake relative to a reference region. In FP-CIT PET, SUVR is calculated as the ratio of the mean standardized uptake value (SUV) in a target region (e.g., striatum, caudate or putamen) to that in a reference region with negligible dopamine transporter density, such as the occipital cortex or cerebellum. Because the reference region reflects nonspecific binding, SUVR serves as a surrogate measure of striatal DAT availability and thus, presynaptic dopaminergic integrity. Normalization to a reference region also reduces variability related to injected dose, body weight, and global tracer delivery, allowing comparisons across subjects and between hemispheres. SUVR values were defined as follows:

SUVR = mean SUV of the target region/mean SUV of the occipital cortex.

Additional PET-derived measures included regional asymmetry indices. Regional asymmetry indicates the disparity in SUVR between the right and left side of each brain region. The formula for the regional asymmetry index is as follows:

Regional asymmetry index = (Left − Right)/(Left + Right) × 100

To obtain a single summary measure of BL nigrostriatal dopaminergic deficit, a study-specific DAT burden score was constructed from bilateral putaminal and striatal ^18^F-FP-CIT uptake values. These regions were selected because quantitative DAT imaging consistently demonstrates reduced striatal, particularly putaminal, uptake in PD and associations between reduced DAT measures and greater motor severity [5,27,28]. This combination was intended to retain explicit representation of putaminal uptake alongside a broader striatal uptake summary. For the present analysis, two BL DAT-derived summary measures were calculated.

First, an overall DAT burden score was computed using the z-standardized uptake values of the right and left putamen and the right and left striatum:

DAT burden score = −mean z (Putamen_Rt, Putamen_Lt, Striatum_Rt, Striatum_Lt).

The sign was reversed so that higher DAT burden scores indicated lower regional ^18^F-FP-CIT uptake and greater dopaminergic deficit. Second, to summarize the lateralized pattern of dopaminergic involvement, a DAT asymmetry score was calculated using predefined asymmetry measures of the putamen, striatum, and caudate:

DAT asymmetry score = mean z (Putamen asymmetry, Striatum asymmetry, Caudate asymmetry).

Regional asymmetry in striatal DAT availability is a well-established feature of PD, and quantitative DAT imaging studies have evaluated asymmetry indices across the putamen, caudate, and overall striatum [28,29,30]. Higher composite scores, therefore, represented greater overall asymmetry in striatal dopaminergic uptake. These DAT-derived measures were used as BL biological markers for comparison with the wearable sensor-derived turning movement-dynamics signature.

### 2.4. Wearable Sensor-Based Gait Assessment

Quantitative gait assessments were performed using wearable IMU. Participants completed a standardized set of gait tasks, including forward and backward straight walking at preferred speed and 360° turning at both preferred and fast speeds [12]. For the preferred- and fast-speed conditions, participants were instructed to perform the task at their usual, self-selected walking speed and at a fast walking speed, respectively. All participants performed the turning tasks in the following standardized, fixed order: forward and backward straight walking, preferred-speed left and right turns, and fast-speed left and right turns. The task order was not randomized. For the forward and backward straight-walking tasks, participants walked along an 8-m walkway, and gait data from the middle 4-m section were analyzed to minimize acceleration and deceleration effects. For the 360° turning task, participants rose from a chair, walked to a cone placed 3 m away, performed a 360° turn around the cone, and then exited the walkway. Turning was performed to both the left and right sides at preferred and fast speeds. Each task was repeated three times, with a 1-min rest interval between trials. All gait tasks were conducted under controlled laboratory conditions to minimize environmental variability. Although gait assessments were conducted longitudinally (BL to final follow-up), the present prognostic analysis included only wearable sensor-derived gait features obtained at BL as predictors. Follow-up gait measurements were used solely to characterize longitudinal changes and were not entered into the prognostic models. This design enabled the evaluation of whether gait characteristics available at BL provided prognostic information on motor progression over approximately 22 months.

**Figure 2:**
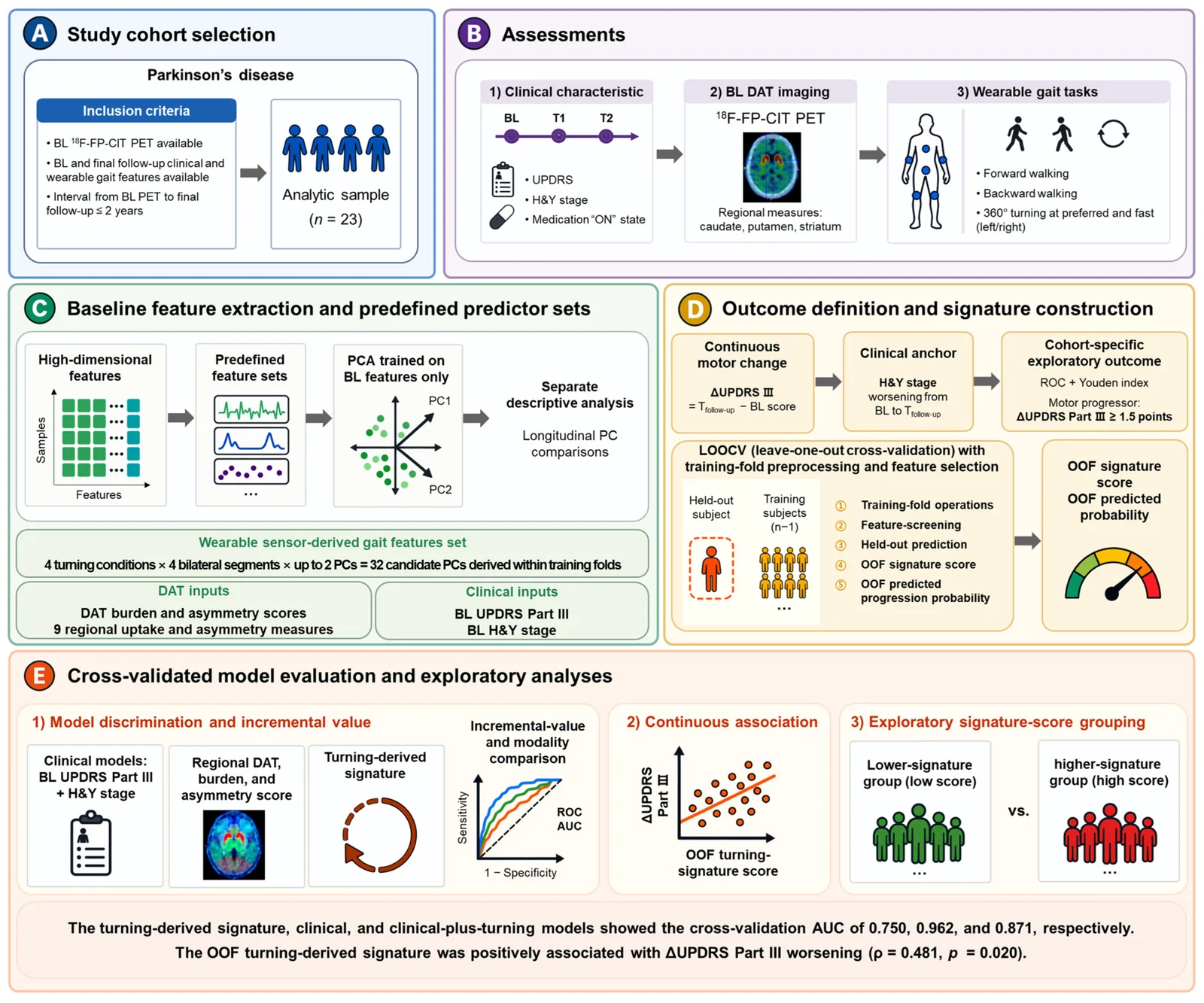
An overview of the analytical workflow. BL: baseline; PET, positron emission tomography; UPDRS, Unified Parkinson’s Disease Rating Scale; H&Y: Hoehn and Yahr; DAT: dopamine transporter; PCA: principal component analysis; ROC: receiver operating characteristic; OOF: out-of-fold; AUC: area under the receiver operating characteristic curve.

Sensors were placed on six anatomical landmarks: the left and right lateral humeral epicondyles (LELB and RELB), left and right lateral thigh (LTHI and RTHI), 10th thoracic vertebra (T10), and posterior superior iliac spine (PSIS). This sensor configuration was designed to capture whole-body movement patterns and gait dynamics across upper-limb, lower-limb, and axial body segments. A schematic illustration of the experimental setup and IMU sensor placement is presented in Figure 2B. The IMU sensors were calibrated before each trial according to the manufacturer’s guidelines. Kinematic data were collected using six IMU sensors (Xsens DOT, Movella Technologies, Enschede, Netherlands) at a sampling frequency of 60 Hz. Gait acquisition followed the laboratory protocol described in our previous study [12]. For forward and backward walking, a central four-step segment was selected for analysis to limit the contribution of gait initiation and termination. For each 360° turning task, the initiation phase was defined as the point at which the angle between the pelvic and mediolateral vectors exceeded 10°. The rotation ended when the two vectors completed a 360° turn, and the 350° turning phase was subsequently analyzed. Extracted sensor-derived features included acceleration- and angular velocity-based measures, such as maximum jerk, angular velocity jerk, mean acceleration, root mean square (RMS) acceleration, gyroscope-derived measures, and magnitude- and irregularity-related features [12,31]. Wearable sensor-derived features were calculated separately for each trial and sensor over the selected task interval. For each participant, assessment visit, task condition, and sensor location, the arithmetic mean of the three trial-level feature values was used in subsequent analyses. Detailed feature definitions and calculation procedures are provided in Supplementary Materials Table S3. Owing to the high number of wearable sensor-derived gait variables, gait features were reduced using principal component analysis (PCA) and summarized at the principal component (PC) or feature-set level rather than reporting descriptive statistics for all individual sensor variables [32,33].

### 2.5. Data Processing

The analysis comprised five main steps: (1) extraction and organization of data, including demographic and clinical assessments, wearable sensor-derived gait features, and BL ^18^F-FP-CIT PET measures; (2) dimensionality reduction in high-dimensional gait features using PCA; (3) definition of a clinically anchored motor progression outcome based on longitudinal changes in UPDRS Part III and H&Y stage from BL to final follow-up; (4) construction of a leakage-controlled, task-specific wearable-derived gait signature using a leakage-reduced leave-one-out cross-validation (LOOCV) framework; and (5) comparison of the cross-validated prognostic performance of BL clinical severity measures, DAT-derived measures, the wearable-derived gait signature, and a model combining the clinical measures with the gait signature (Figure 2).

Wearable sensor-derived gait features included acceleration- and angular velocity-based metrics obtained across tasks and body segments. To reduce dimensionality while preserving task- and segment-specific movement characteristics, gait features were organized by task, sensor location, and feature family. No imputation was required as the final analytic sample contained no missing values. Within each LOOCV training fold, gait features were standardized using the training-set means and standard deviations, after which PCA was fitted exclusively to the training data. PCs were retained sequentially until the cumulative explained variance reached 80%, with a maximum of two components retained for each task–segment–feature-family combination. This procedure generated low-dimensional gait representations while minimizing redundancy among highly correlated sensor-derived variables [32]. For descriptive reporting of explained variance and feature loadings in the Supplementary Materials (Tables S4 and S5), PCA was additionally performed using the complete BL analytic dataset; these full-sample PCA results were not used to generate the cross-validated prognostic predictions.

Although multiple gait tasks were collected, the main wearable-derived gait analysis focused on a limb-based turning movement-dynamics feature family. This family included four 360° turning conditions—preferred- and fast-speed turns to the left and right—and four bilateral limb segments: the left and right elbows and the left and right thighs. For each task–segment combination, the input variables comprised the RMS and sample entropy of linear acceleration and angular velocity, resulting in 64 raw BL features. RMS quantified the overall magnitude of the sensor signal, whereas sample entropy quantified its temporal irregularity or predictability [34,35]. Accordingly, this feature family was not considered a conventional measure of stride-to-stride variability. Within each outer LOOCV training fold, the raw features were standardized and reduced using PCA. Up to two PCs were retained for each task–segment combination according to the prespecified cumulative explained-variance criterion, producing up to 32 candidate PCs. The resulting signature is therefore referred to as the “limb-focused turning movement-dynamics signature,” or the “turning-derived signature” in abbreviated form. T10 and PSIS were not excluded because of missing data, recording quality, or PCA-based rejection; rather, they fell outside the limb-focused candidate family used for the primary exploratory analysis.

Longitudinal motor progression was quantified using the change in UPDRS Part III score from BL to final follow-up:

ΔUPDRS Part III = UPDRS Part III_final − UPDRS Part III_BL (1)

To derive a clinically anchored binary motor progression outcome, worsening of H&Y stage from BL to final follow-up was used as the external clinical anchor. Receiver operating characteristic (ROC) analysis was performed using ΔUPDRS Part III to identify participants with H&Y stage worsening, and the optimal threshold was determined using the Youden index.

A leakage-reduced LOOCV framework with training-only outcome-informed feature selection was used to construct the BL turning-derived signature and reduce optimistic bias associated with feature selection [36]. In each iteration, one participant was held out as the test case, and feature screening and signature construction were performed exclusively using the remaining participants [37]. Within the training set, each candidate turning-derived signature was evaluated for its association with continuous ΔUPDRS Part III using a linear regression model adjusted for age, sex, and BL UPDRS Part III score as follows:

ΔUPDRS Part III = β0 + β1 (gait PCs) + β2 (age) + β3 (sex) + β4 (BL UPDRS Part III) (2)

Candidate PCs were ranked based on their training-set *p*-values, and up to five candidates with *p* values < 0.05 were retained. If no candidate met this criterion, the five candidates with the lowest *p*-values were retained as a rank-based fallback. Feature-selection recurrence was summarized as the number of LOOCV folds in which each task–sensor–PC label was selected, divided by 23, and then multiplied by 100. Because PCA was refitted within each training fold, matching labels identified the same task, sensor location, and component order, but not necessarily identical loading vectors. This selection rule was applied consistently to the turning-derived and regional DAT signatures.

Selected features were standardized using the mean and standard deviation estimated exclusively from the corresponding training set. Because individual gait features could be positively or negatively associated with subsequent motor worsening, their directions were aligned based on the sign of the training-set regression coefficient. For feature j, the direction-aligned standardized value was calculated as:

(3) $$z_{ij}=\left(\frac{x_{ij}-\mu_{j,train}}{\sigma_{j,train}}\right)\times sign(\beta_{j,train})$$

where $x_{ij}$ denotes the value of feature *j* for participant *i*, $\mu_{j,train}$ and $\sigma_{j,train}$ are the mean and standard deviation estimated from the training set, respectively, and $\beta_{j,train}$ is the regression coefficient relating feature *j* to ΔUPDRS Part III within the training set. Accordingly, features positively associated with motor worsening retained their original standardized direction, whereas negatively associated features were sign-reversed. A study-specific fold-level turning-derived signature was then calculated as the unweighted mean of the *K* selected and direction-aligned features:

(4) $${Turning-derived\;signature}_{i}=\frac{1}{K}\sum_{j=1}^{K}z_{ij}$$

where *K* denotes the number of features selected within the corresponding training fold (maximum of five features). This procedure placed all selected features on a common scale and common directional interpretation before aggregation.

To provide a modality-balanced exploratory comparison, an analogous regional DAT signature was constructed from nine candidate measures: right and left caudate, putamen, and striatum uptake values and caudate, putamen, and striatum asymmetry indices. Within each outer LOOCV training fold, the regional DAT candidates were screened using the same age-, sex-, and BL UPDRS Part III-adjusted regression model applied to the turning-derived candidates. The candidates were then selected, standardized, direction-aligned, and averaged according to the same training-fold procedure used for the turning-derived signature. PCA was not applied to the regional DAT candidates because they represented prespecified regional imaging measures rather than high-dimensional sensor-derived features.

The training-derived feature transformations, PCA loadings, feature directions, and signature-construction rules were applied to the held-out participant to generate one out-of-fold (OOF) turning-signature score per participant. The same training-derived procedures were applied to the participants within the corresponding training fold to calculate their fold-specific signature scores.

To assess the prognostic information provided by BL clinical severity, the turning-derived signature, and the equivalently constructed regional DAT signature, six logistic regression models were evaluated: (1) BL UPDRS Part III alone; (2) BL H&Y stage alone; (3) BL UPDRS Part III and H&Y stage together, designated as the clinical model; (4) the regional DAT signature alone; (5) the turning-derived signature alone; and (6) BL UPDRS Part III and H&Y stage combined with the turning-derived signature, designated as the combined model. H&Y stage was entered as an ordinal numeric predictor. Within each LOOCV iteration, predictor standardization and logistic regression model fitting were performed using only the training participants. All logistic regression models used L2 regularization and class-balanced weights. The probability of study-defined motor progression for held-out participant *i* in fold *f* was calculated as:

(5) $$\hat{p}ᵢf\;=\;1/[1\;+\;exp\{-(\hat{\beta }0f\;+\;zᵢfᵀ\hat{\beta }f)\}]$$

where z_i_*f* represents the predictor values standardized using the means and standard deviations of the corresponding training fold, whereas *β*0*f* and *βf* denote the intercept and regression coefficients estimated exclusively from that training fold. For the turning-signature model, z_i_*f* consisted of the standardized fold-specific turning-signature score. For the clinical model, it consisted of standardized BL UPDRS Part III and H&Y stage values, whereas the combined model included both clinical predictors and the turning-signature score. The fitted training-fold model was then applied to the held-out participant, who had not contributed to predictor standardization or model fitting [38]. Aggregating these held-out probabilities across all LOOCV iterations yielded one OOF predicted probability for each participant.

Model discrimination was quantified using the area under the receiver operating characteristic curve (AUC) calculated from the aggregated OOF probabilities. The incremental prognostic contribution of the turning signature was evaluated by comparing the cross-validated AUC of the clinical model with that of the combined model. The 95% confidence intervals (CIs) for individual AUCs and the paired difference in AUC were estimated using 10,000 stratified bootstrap replicates. The paired AUC difference was additionally evaluated using a prediction-swap permutation test with 20,000 permutations. For exploratory risk stratification, participants were divided into lower- and higher-signature groups using the median OOF signature score; this threshold was not considered a clinically validated prognostic cut-off.

### 2.6. Statistical Analysis

Continuous variables were assessed for normality using the Shapiro–Wilk test and summarized as mean and standard deviation, while categorical variables are presented as counts in Table 1. To assess whether variability in follow-up duration could have influenced the magnitude of longitudinal motor change, the association between the BL-to-final follow-up interval and ΔUPDRS Part III was evaluated using Spearman’s rank correlation. Follow-up duration was also compared between study-defined motor progressors and non-progressors using a two-sided Mann–Whitney U test.

Associations of BL DAT-derived measures and the wearable turning-derived signature with continuous motor progression (ΔUPDRS Part III) were examined using OLS regression models adjusted for age, sex, and BL UPDRS Part III. For exploratory feature-family analyses, the Benjamini–Hochberg FDR correction was applied to feature-level association tests within each predefined feature family and across all screened gait features. The association between the OOF turning-derived signature and ΔUPDRS Part III was further assessed using Spearman’s rank correlation coefficient. Differences in ΔUPDRS Part III between the lower- and higher-risk signature groups were evaluated using the two-sided Mann–Whitney U test.

Prognostic discrimination was evaluated using ROC analysis. For all models, ROC curves and AUCs were calculated from OOF predicted probabilities aggregated across all outer LOOCV iterations. The predefined DAT burden and DAT asymmetry scores were each entered separately as the sole predictor in a logistic regression model. For these two models, predictor standardization and logistic regression fitting were performed within each training fold, and the fitted model generated a predicted probability for the held-out participant. The 95% CIs for individual AUCs were estimated using 10,000 stratified bootstrap replicates. For the clinical, regional DAT signature, turning-derived signature, and combined models, statistical significance of the internally cross-validated AUCs was evaluated using 2000 full-pipeline outcome permutations. In each permutation, continuous ΔUPDRS Part III and its corresponding binary motor-progression label were permuted together across participants to preserve their paired relationship. All outcome-dependent procedures, including training-fold feature screening, direction alignment, signature construction, predictor standardization, logistic regression fitting, and held-out probability estimation, were repeated within the outer LOOCV framework. The empirical one-sided permutation *p* value was calculated as (b + 1)/(B + 1), where b denotes the number of permuted AUCs greater than or equal to the observed AUC and B denotes the total number of permutations.

Prespecified paired differences in cross-validated AUC, including the comparison between the clinical and combined models and that between the turning-derived and regional DAT signatures, were evaluated using 95% CIs derived from 10,000 stratified bootstrap replicates. Statistical significance of each paired AUC difference was additionally evaluated using a two-sided prediction-swap permutation test with 20,000 permutations. For all models, accuracy, balanced accuracy, F1 score, sensitivity, specificity, positive predictive value, and negative predictive value were calculated by classifying participants with an OOF predicted probability ≥ 0.50 as progressors. This threshold applied to predicted probabilities, not to the original DAT composite scores.

All tests were two-sided, with *p* < 0.05 considered statistically significant unless otherwise specified. Statistical analyses were performed using SPSS Statistics version 21.0 (IBM Corp., Armonk, NY, USA) and Python version 3.10 with NumPy, pandas, SciPy, statsmodels, and scikit-learn.

## 3. Results

### 3.1. Dimensionality Reduction in Limb-Focused Turning Movement-Dynamics Signature

**Figure 3:**
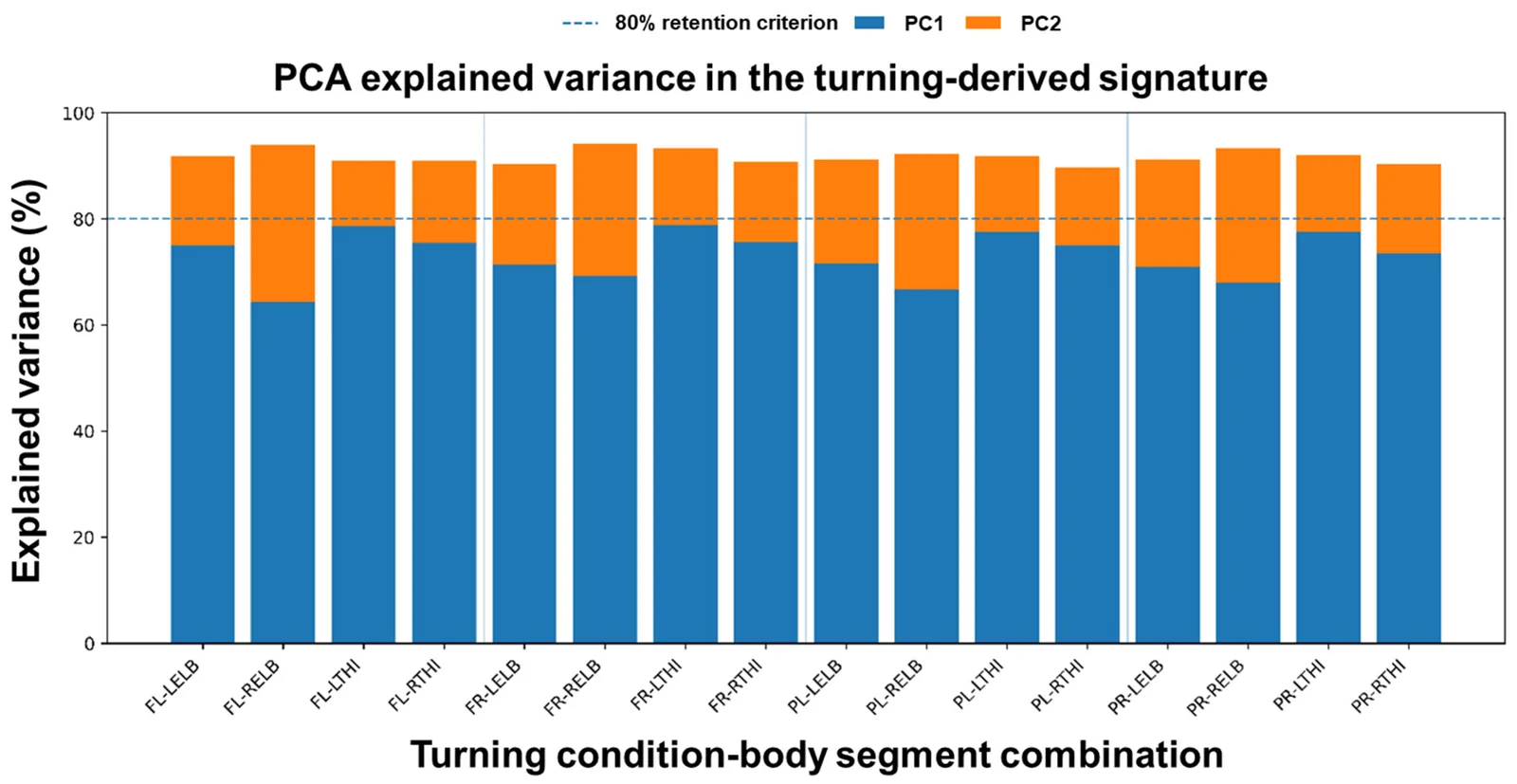
Principal component analysis of the limb-focused turning movement-dynamics signature. Stacked bars indicate the percentage of variance explained by PC1 and PC2 across the 16 combinations of four 360° turning conditions and four bilateral body segments. The dashed horizontal line represents the prespecified 80% cumulative explained-variance criterion. Two PCs were retained for each combination. The PCA shown in this figure summarizes the full BL analytic cohort for descriptive interpretation; in the prognostic analyses, standardization and PCA were refitted separately within each LOOCV training fold. FL: Left turning at fast speed; FR: Right turning at fast speed; PL: Left turning at preferred speed; PR: Right turning at preferred speed; LELB: Left lateral humeral epicondyles; RELB: Right lateral humeral epicondyles; LTHI: Left lateral thigh; RTHI: Right lateral thigh.

For descriptive characterization of the complete BL analytic dataset, PCA effectively reduced the dimensionality of sensor-derived gait features while preserving most variance within each task–segment feature set. For the primary limb-focused turning movement-dynamics signature, 64 raw turning-derived signatures from 16 task–segment combinations were reduced to 32 PC features (Figure 3). Two PCs were retained for each combination, explaining 89.6–94.0% of the original variance (mean, 91.7%). PC1 accounted for 64.4–78.8% of the variance (mean, 73.1%) and was characterized by the largest absolute loading from acceleration RMS, whereas PC2 accounted for an additional 12.2–29.5% (mean, 18.6%) and was predominantly characterized by gyroscope RMS or gyroscope sample entropy. Detailed explained-variance estimates and feature loadings are provided in Supplementary Materials Tables S4 and S5. These full-sample PCA results were used only to describe the component structure; for prognostic evaluation, PCA was refitted independently within each LOOCV training fold.

Feature selection across the 23 LOOCV folds was concentrated in elbow-derived components, particularly during fast turning (Supplementary Materials Table S6). The right-elbow PC2 during fast right turns was selected in 21 folds (91.3%), followed by the right-elbow PC2 during fast left turns in 19 folds (82.6%). The left-elbow PC2 during fast left turns and the left-elbow PC1 during fast right turns were each selected in 17 folds (73.9%). Two preferred-speed elbow-derived PC2 features were each selected in 10 folds (43.5%). In the descriptive BL PCA loading structure, the three frequently selected PC2 features were characterized most strongly by angular-velocity sample entropy or angular-velocity RMS, whereas the fast-right-turn left-elbow PC1 had its largest absolute loading on acceleration RMS. Thus, the recurrently selected features represented a combination of upper-limb angular-velocity magnitude, temporal irregularity, and acceleration magnitude during turning rather than a single conventional variability measure.

### 3.2. Motor Progression Outcome

**Figure 4:**
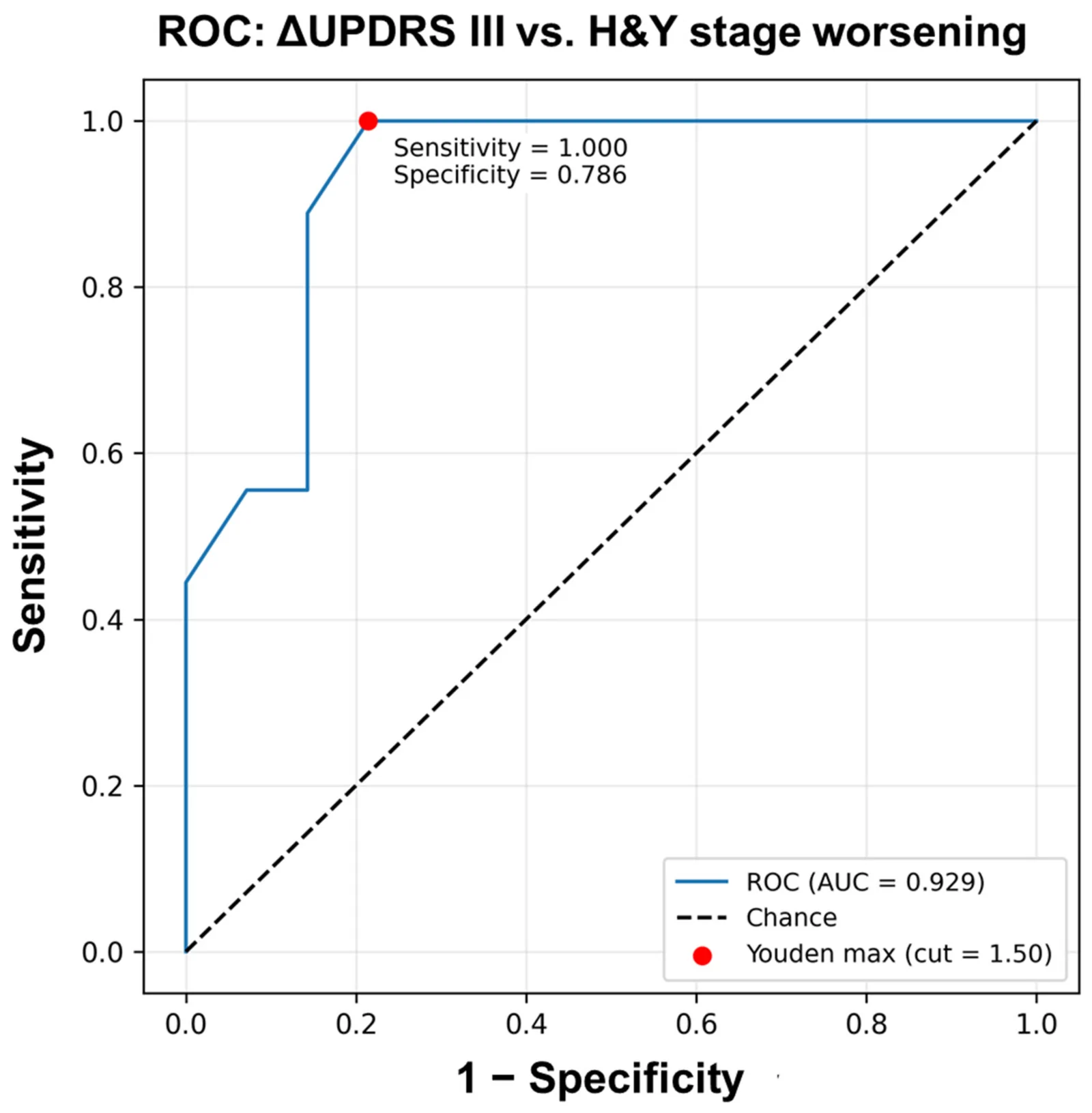
Receiver operating characteristic analysis for deriving the cohort-specific, H&Y-anchored exploratory motor progression threshold. Worsening of the Hoehn and Yahr (H&Y) stage from baseline (BL) to final follow-up was used as the clinical anchor. Receiver operating characteristic (ROC) analysis was performed using Δ Unified Parkinson’s Disease Rating Scale Part III (ΔUPDRS Part III), calculated as the final follow-up score minus the BL score, with positive values indicating worsening. The optimal threshold was determined using the Youden index. The marked point indicates the Youden index cut-off, and the dashed diagonal line represents chance-level discrimination.

Longitudinal motor progression was quantified by the change in UPDRS Part III score from BL to final follow-up. To define a clinically anchored binary motor progression outcome, an increase in H&Y stage from BL to final follow-up served as the clinical anchor. ROC analysis was conducted using ΔUPDRS Part III as the candidate progression measure. In this cohort, the Youden index identified ΔUPDRS Part III ≥ 1.5 as the optimal cohort-specific exploratory threshold for classifying study-defined motor progressors. This threshold demonstrated high agreement with H&Y stage increase, with a sensitivity of 1.000, specificity of 0.786, and AUC of 0.929 (Figure 4). Based on this H&Y-anchored, cohort-specific exploratory threshold, 12 participants were classified as study-defined motor progressors and 11 as non-progressors, corresponding to a motor progressor rate of 52.2%.

### 3.3. Cross-Validated Discrimination of BL Clinical, DAT-Derived, and Turning-Signature Models

BL motor severity differed substantially between the study-defined progression groups. Non-progressors had higher BL UPDRS Part III scores (38.55 ± 11.86 vs. 17.50 ± 6.24) and higher BL H&Y stages (2.18 ± 0.40 vs. 1.42 ± 0.51) than progressors.

In the discrimination analyses (Table 2), BL UPDRS Part III alone showed an internally cross-validated AUC of 0.947, whereas BL H&Y stage alone showed an AUC of 0.659. The clinical model combining BL UPDRS Part III and H&Y stage yielded an AUC of 0.962 (full-pipeline permutation *p* < 0.001). The predefined DAT burden and DAT asymmetry measures showed limited discrimination, with AUCs of 0.470 and 0.462, respectively.

When the training-fold outcome-informed procedure used for the turning-derived signature was applied equivalently to the nine regional DAT candidates, the resulting regional DAT signature showed chance-level OOF discrimination, with an AUC of 0.500 (full-pipeline permutation *p* = 0.441). The fully nested turning-derived signature yielded an AUC of 0.750 (full-pipeline permutation *p* = 0.020). Although the turning-derived signature showed a 0.250 higher observed AUC than the regional DAT signature, the paired difference was not statistically significant (paired prediction-swap *p* = 0.122). Adding the turning-derived signature to BL UPDRS Part III and H&Y stage did not improve cross-validated discrimination. The combined clinical-plus-turning model yielded an AUC of 0.871 (full-pipeline permutation *p* = 0.002), corresponding to a paired ΔAUC of −0.091 relative to the clinical model (paired prediction-swap *p* = 0.015). Thus, although the turning-derived signature showed exploratory standalone discrimination and a higher observed AUC than the regional DAT signature, it did not demonstrate statistically significant superiority over the regional DAT signature or incremental prognostic value beyond BL clinical severity in this cohort.

### 3.4. Association Between Risk-Aligned Turning-Derived Signature and Motor Progression

**Figure 5:**
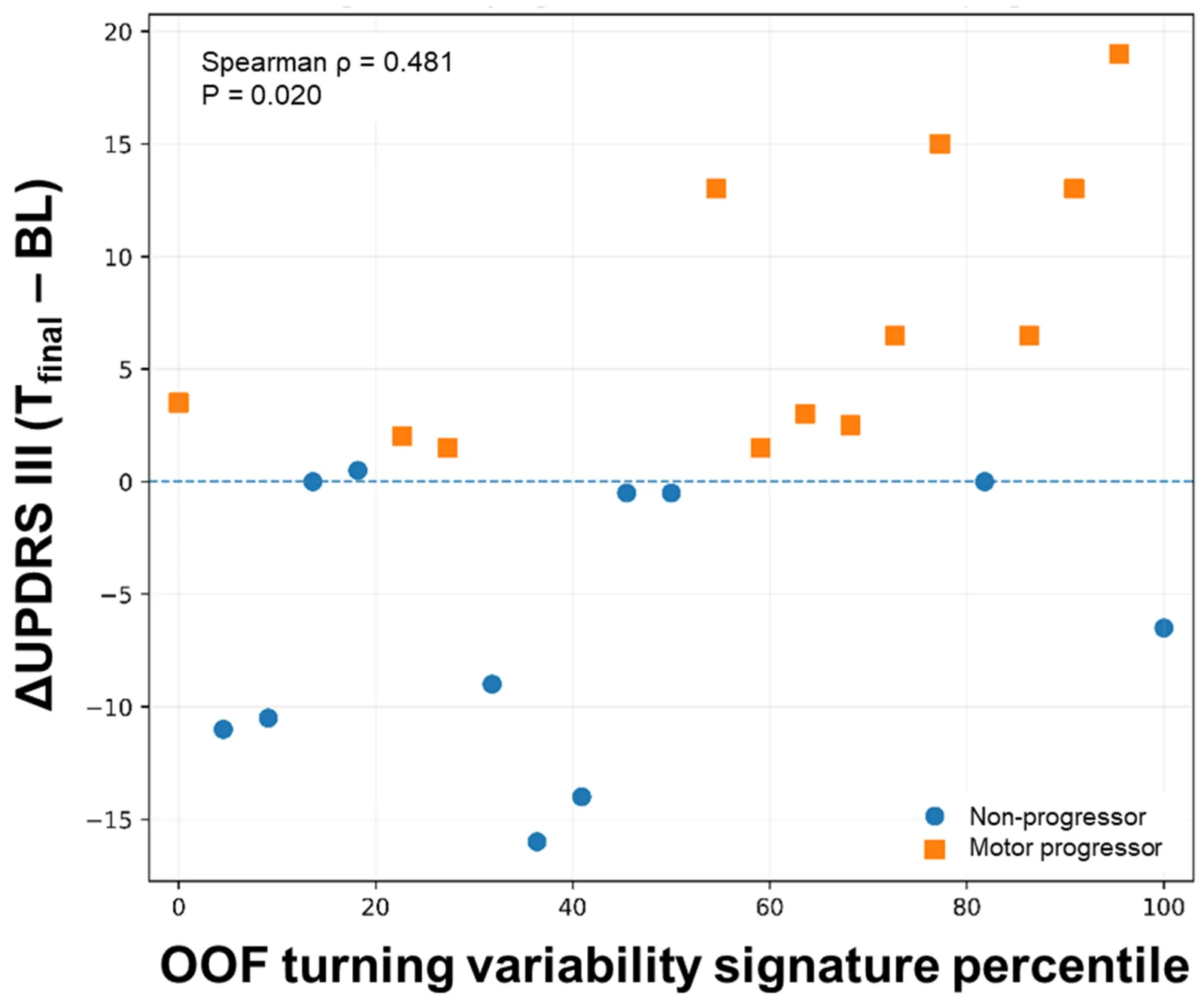
Association between the risk-aligned out-of-fold turning-derived signature and motor progression. The x-axis represents the risk-aligned out-of-fold (OOF) limb-focused turning movement-dynamics signature. For visualization, continuous OOF signature scores were rank-transformed to percentiles; this monotonic transformation does not alter Spearman’s rank correlation. The y-axis represents ΔUnified Parkinson’s Disease Rating Scale Part III (ΔUPDRS Part III), calculated as the final follow-up score minus the baseline (BL) score. Positive values indicate motor worsening, whereas negative values indicate lower UPDRS Part III scores at follow-up. Each point represents one participant and is colored according to study-defined motor progression status. The solid fitted line is provided for visual guidance, and the horizontal dashed line indicates no change from BL. Higher OOF turning-signature scores were associated with greater increases in UPDRS Part III.

To further evaluate the association between the turning-derived signature and the magnitude of subsequent motor worsening, we examined the relationship between the risk-aligned OOF turning-derived signature and ΔUPDRS Part III. Higher risk-aligned OOF signature scores correlated with greater increases in UPDRS Part III from BL to final follow-up. Spearman’s rank correlation analysis indicated a moderate positive association between the turning-derived signature and ΔUPDRS Part III (ρ = 0.481, *p* = 0.020; Figure 5).

### 3.5. Motor Progression According to Low- and High-Risk Turning-Derived Signature Groups

**Figure 6:**
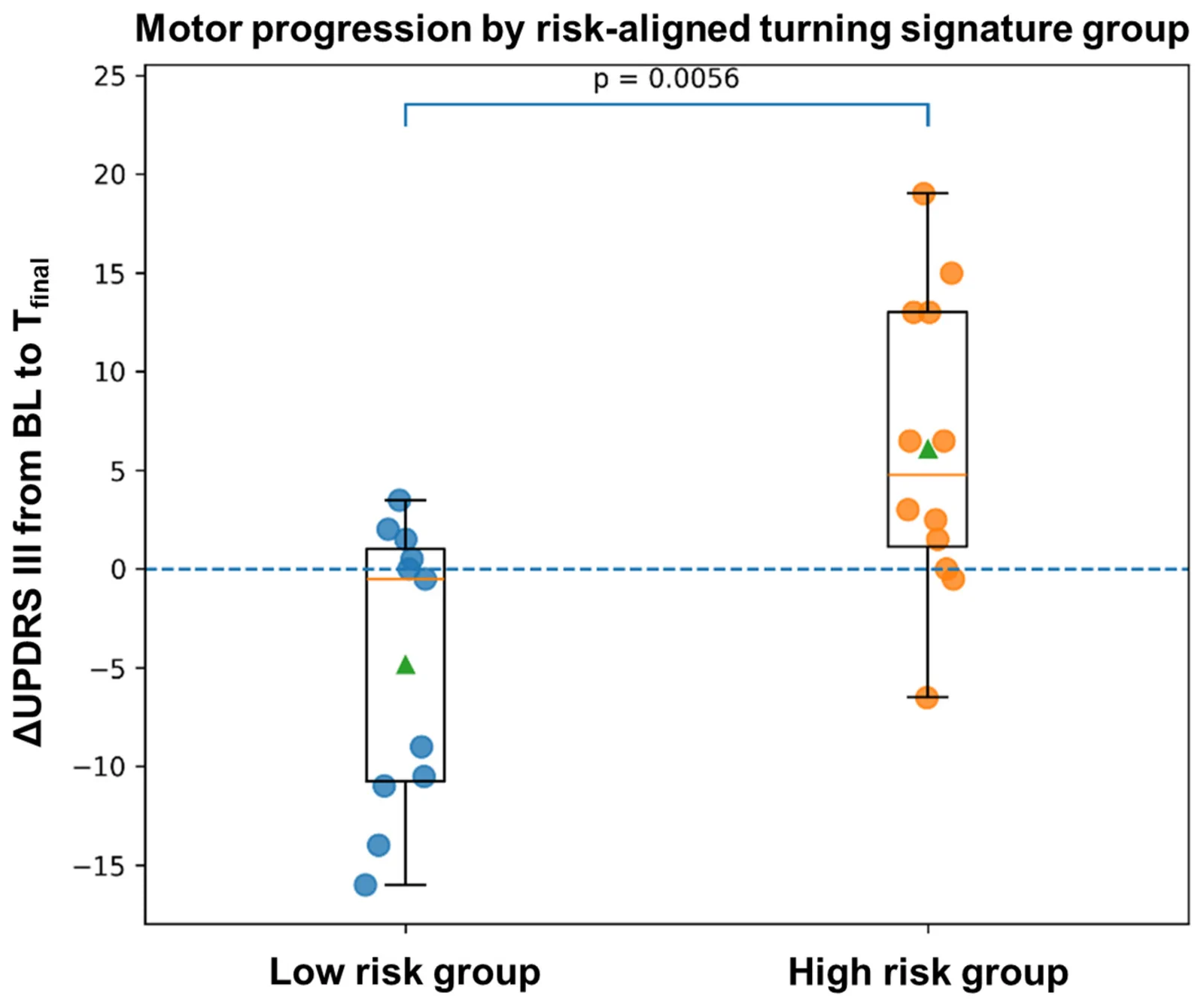
Comparison of motor progression according to the baseline turning-derived signature group. Participants were divided into lower- and higher-signature groups using the median risk-aligned out-of-fold (OOF) turning-signature score. Individual circles represent participant-level ΔUnified Parkinson’s Disease Rating Scale Part III (ΔUPDRS Part III), calculated as the final follow-up score minus the baseline (BL) score. Positive values indicate motor worsening, whereas negative values indicate lower motor scores at follow-up. Box plots show the median and interquartile range, whiskers indicate values within 1.5 times the interquartile range, and triangles indicate group means. The horizontal dashed line represents no change from BL. The median split was used only for exploratory visualization and does not represent a clinically validated prognostic cut-off.

For exploratory risk stratification, individuals with PD were divided into low- and high-progression-risk groups based on the median of the risk-aligned OOF turning-derived signature score (Figure 6). The low-risk group (*n* = 11) showed no average worsening of motor symptoms by final follow-up; the mean ΔUPDRS Part III was −4.86, with a median change of −0.50. The motor progressor rate in this group was 27.3%, indicating that most patients classified as low-risk based on the BL turning-derived signature remained clinically stable or improved over follow-up. In this group, 7 patients (63.6%) showed stable or improved UPDRS Part III scores, while 4 patients (36.4%) experienced an increase in UPDRS Part III scores. H&Y stage worsening was observed in only 1 patient (9.1%). In contrast, the high-risk group (*n* = 12) exhibited clear motor worsening by final follow-up. The mean ΔUPDRS Part III was +6.08, with a median change of +4.75. The motor progressor rate in this group was 75.0%, indicating that most PD patients classified as high-risk based on the BL turning-derived signature subsequently met the H&Y-anchored motor progression criterion. H&Y stage worsening was observed in 8 patients (66.7%) in this group. The difference in ΔUPDRS Part III between the low- and high-risk groups was significant according to the Mann–Whitney U test (*p* = 0.0056).

## 4. Discussion

The present exploratory longitudinal study investigated whether BL DAT-derived measures and wearable sensor-derived turning characteristics could provide prognostic information regarding subsequent study-defined motor progression in individuals with PD. Three principal findings emerged. First, BL clinical severity showed strong discrimination for the study-defined progression outcome, and adding the turning-derived signature did not improve discrimination beyond that achieved by the clinical model. Second, the predefined DAT burden and asymmetry scores and the equivalently constructed regional DAT signature showed limited discrimination. The turning-derived signature showed higher observed standalone discrimination than the regional DAT signature, although the paired difference was not statistically significant. Third, the risk-aligned OOF turning-derived signature was positively associated with the magnitude of subsequent change in UPDRS Part III, and exploratory signature-based groups showed different motor trajectories. Collectively, these findings suggest that the turning-derived signature captures clinically relevant information [14], but should not be interpreted as providing incremental value beyond BL clinical severity or as being statistically superior to DAT imaging.

The marked BL difference between progressors and non-progressors is central to the interpretation of these findings. Non-progressors had substantially higher BL UPDRS Part III scores and H&Y stages than progressors. Consistent with this imbalance, BL UPDRS Part III alone showed a cross-validated AUC of 0.947, and the clinical model combining BL UPDRS Part III and H&Y stage yielded an AUC of 0.962. Adding the turning signature did not improve discrimination, with the combined model yielding an AUC of 0.871 and a paired ΔAUC of −0.091 relative to the clinical model. Although the combined model produced favorable classification metrics at the fixed probability threshold of 0.5, these single-threshold results do not override the absence of improvement in the primary threshold-independent AUC analysis [39]. Because the progression outcome was defined using the difference between final and BL UPDRS Part III scores, the association between BL severity and the resulting change-based classification may partly reflect mathematical coupling and regression to the mean [40,41]. Participants with relatively high BL scores had greater opportunity to show stable or lower scores at follow-up, whereas those with relatively low BL scores had greater opportunity to show increases [42]. Medication-state effects and within-participant measurement variability may also have contributed [43,44]. Therefore, BL severity may account for a substantial proportion of the observed group separation, and neither the high discrimination of the clinical model nor the standalone discrimination of the turning signature should be interpreted as externally validated prognostic performance [45].

The comparison between the imaging and wearable modalities also requires cau-tious interpretation. When an equivalent training-fold selection and signature-construction procedure was applied to the regional DAT measures, the regional DAT signature showed chance-level discrimination, whereas the turning-derived signature showed higher observed discrimination. However, the paired AUC difference between these signatures was not statistically significant. Moreover, the significant permutation result for the turning-derived signature indicates that its cross-validated AUC was unlikely under permuted outcome assignments; it does not demonstrate that the turning-derived signature was statistically superior to the regional DAT signature. The observed difference may reflect the closer functional proximity of turning performance to the clinical motor outcome, differences in the number and nature of candidate predictors, or sampling variability in this small cohort [46]. Accordingly, the present findings suggest that BL turning-derived characteristics may contain complementary prognostic information that was not captured by the DAT measures evaluated in this exploratory cohort [47].

The limited discrimination of the BL DAT-derived measures may reflect the distinction between dopaminergic pathology and its subsequent functional expression. DAT imaging provides an in vivo marker of presynaptic nigrostriatal dopaminergic integrity and has been shown to be associated with motor severity and disease progression [6,39]. In this study, PET imaging was acquired only at BL; consequently, longitudinal comparisons concern clinical trajectories following BL imaging rather than changes in DAT availability itself. The broadly overlapping BL DAT burden and asymmetry scores, despite subsequent divergence in H&Y and UPDRS Part III trajectories, suggest that a single BL DAT summary may be insufficient to estimate how rapidly dopaminergic pathology will become functionally expressed [47]. Functional progression may depend on the regional distribution of striatal degeneration, compensatory capacity, medication response, and involvement of non-dopaminergic systems [40]. Moreover, the DAT burden and asymmetry scores used in this study summarize regional uptake patterns and may obscure specific spatial gradients associated with progression [6]. Thus, the present findings indicate limited discrimination from these study-specific BL DAT summary measures for the cohort-specific progression outcome. These findings should not be interpreted as evidence of limited biological relevance of DAT imaging or as establishing the superiority of the turning signature, particularly because BL clinical severity strongly discriminated the progression groups.

In contrast, turning may provide a functional challenge that reveals the integrated consequences of these pathological and compensatory processes. Turning requires rapid modification of an ongoing motor program, asymmetric control of limbs, intersegmental coordination, axial reorientation, and continuous regulation of dynamic stability [15,48,49]. These functions are impaired in PD, and longitudinal studies have shown progression-related changes in turning duration, step organization, variability, and regularity [14,50]. The task- and segment-specific turning-derived signature observed in this study may therefore reflect not only the extent of dopamine loss but also how effectively the motor system organizes movement under increased control demands [14,51]. This may explain why patients with a similar degree of BL dopaminergic deficit exhibit different turning patterns and subsequently different motor trajectories [52,53]. During follow-up, the levodopa-equivalent daily dose increased in both groups, with a descriptively larger mean increase among progressors than among non-progressors (+101.6 vs. +19.5 mg/day). However, final mean doses were nearly identical and interindividual variability substantial. Treatment may have been intensified in response to emerging motor symptoms, but the timing and clinical indications for individual medication adjustments were not examined. In addition, individual pharmacokinetics, motor fluctuations, and the long-duration response to dopaminergic treatment may have affected clinical performance even when assessments were conducted within a similar post-dose interval [43]. Because medication exposure and dose-to-assessment timing were not modeled as time-varying covariates, we could not determine the extent to which medication response contributed to the observed turning-derived associations or longitudinal changes in UPDRS Part III [44]. However, because the turning signature did not improve discrimination beyond BL UPDRS Part III and H&Y stage, these mechanistic interpretations should be regarded as hypothesis-generating rather than as evidence of an independent prognostic contribution.

In addition, movement-dynamics in PD can reflect inconsistent scaling, altered automaticity, impaired intersegmental coordination, or compensatory control, and its clinical significance may depend on the gait task and body segment involved [54]. Their interpretation depended on the turning condition, body segment, and component-loading structure (see Supplementary Materials Tables S4 and S5). Since the turning-derived signature PCs combined RMS- and sample entropy-based measures, they captured multivariate patterns of movement magnitude and temporal irregularity rather than a unidirectional measure of variability [55]. This is consistent with evidence that PD-related turning characteristics vary according to turning speed, body segment, and gait domain [50]. The contribution of bilateral elbow and thigh features may reflect disruption of interlimb coordination during whole-body reorientation, consistent with reports of altered arm-swing magnitude, asymmetry, and magnitude- and irregularity-related component in early or prodromal PD [49,56,57].

Accordingly, DAT imaging and wearable-derived turning assessment characterize different aspects of PD. DAT imaging reflects the underlying nigrostriatal dopaminergic substrate [2,6], whereas turning measures may reflect the functional expression of motor impairment, compensatory control, and broader network-level dysfunction [14,49]. However, the present results do not demonstrate that the turning signature provides incremental prognostic information beyond routine BL clinical severity. Moreover, the observed difference between the turning and DAT models should be interpreted descriptively because the turning signature was constructed from multiple candidate features, whereas the DAT burden and asymmetry measures were predefined composites. Future studies should be aimed at evaluating imaging, clinical, and wearable measures using equivalent modeling procedures, prespecified signatures, and external validation to determine whether their integration improves prognostic assessment [12,58,59].

The continuous and categorical analyses provided exploratory descriptions of the relationship between the turning-derived signature and subsequent motor change but should not be regarded as independent validation. The risk-aligned OOF signature was positively associated with ΔUPDRS Part III, indicating that higher BL risk-signature values were associated with greater subsequent motor worsening. This finding aligns with evidence that quantitative gait and turning characteristics may serve as digital markers of PD severity, progression, and prognosis [14,49]. This relationship should be interpreted as a graded association rather than a deterministic prediction, as PD progression is heterogeneous and unlikely to be fully explained by a single BL motor assessment [47,60]. Consistent with this interpretation, the higher-risk signature group showed a median ΔUPDRS Part III of +4.75 compared with −0.50 in the lower-risk group and contained a larger proportion of motor progressors. However, the median split was used only for exploratory visualization and stratification and should not be considered a clinically validated prognostic threshold [61]. The principal finding remains the continuous relationship between the turning-derived signature and subsequent motor change.

Motor progression in this study was defined using H&Y stage worsening as a clinical anchor. H&Y stage worsened in 39.1% of 23 participants, remained unchanged in 56.5%, and improved in 4.3% between BL and final follow-up [56]. Progressors showed mean increases in H&Y stage (+0.75) and UPDRS Part III (+7.25), while non-progressors exhibited little mean H&Y change (−0.09) and a reduction in UPDRS Part III (−6.14). Furthermore, non-progressors were more impaired at BL than progressors in both H&Y stage (2.18 vs. 1.42) and UPDRS Part III (38.55 vs. 17.50). Changes in other clinical and physical outcomes varied and did not uniformly align with the H&Y-anchored group trajectories (see Supplementary Materials Table S2), indicating that the classification primarily captured the anchored motor dimension rather than generalized deterioration across all disease domains [62]. However, H&Y is an ordinal, nonlinear, and relatively coarse staging system, heavily weighted toward postural instability, and does not fully capture the broader spectrum of motor and non-motor disability in PD [63]. Accordingly, the identified ΔUPDRS Part III threshold of ≥1.5 should be viewed as a cohort-specific, H&Y-anchored progression criterion, not as a general minimal clinically important difference or as an established clinical threshold for motor progression. Published estimates of clinically important motor change are larger, though they vary by scale, study population, clinical anchor, medication state, and direction of change. For the UPDRS motor score, Shulman et al. reported a minimal clinically important difference of approximately 2.5 points [64]. For MDS-UPDRS Part III, Horváth et al. reported an anchor-based estimate of approximately +4.63 points for minimal clinically relevant worsening [65]. Independent validation of the progression definition and turning signature in a larger cohort with more balanced BL severity is therefore required before clinical application.

Several limitations should be considered. First, the small sample size and absence of an independent validation cohort limit the generalizability and precision of the prognostic estimates, as reflected by the wide CI. Selection based on follow-up eligibility, data completeness, and technical quality may have produced a sample that was not representative of the source cohort. In particular, participants with eligible longitudinal assessments and usable clinical and wearable data may differ from those excluded with respect to BL severity, mobility, or subsequent disease course. However, its direction and the magnitude of any resulting selection bias cannot be determined from the present analyses. Internal cross-validation does not resolve this concern because both model development and evaluation were conducted within the selected analytic cohort. The findings should therefore be regarded as exploratory. Second, the turning-derived signature was derived using study-specific feature selection and aggregation procedures. Although fixing the number of selected features to five in every LOOCV fold improved the consistency of score construction, the procedure remains exploratory and requires external replication. Third, the focused signature was restricted to bilateral elbow and thigh features, precluding direct comparison with axial or whole-body turning characteristics. Fourth, all gait and clinical assessments were conducted in the medication ON state. Although an approximately standardized post-dose interval was used, individual medication responsiveness, motor fluctuations, and treatment adjustments during follow-up were not explicitly modeled. Consequently, the medication-independent contribution of the turning-derived signature to subsequent motor change could not be determined. Fifth, DAT imaging was available only at BL, preventing assessment of whether longitudinal dopaminergic decline paralleled subsequent clinical and digital motor changes. The DAT burden score also combined anatomically overlapping putaminal and overall striatal measures, thereby giving greater weight to putaminal binding. Because the putamen is part of the striatum, these measures are anatomically overlapping, and their joint inclusion introduces redundancy and implicitly gives additional weight to putaminal uptake. This study-specific weighting may influence its performance and should not be assumed to provide an optimal summary of regional DAT loss. The resulting score should therefore be interpreted as a study-specific exploratory composite of reduced DAT uptake rather than a validated measure of global dopaminergic deficit. Sixth, outcome-informed feature selection remained vulnerable to instability and overfitting because each LOOCV training set contained only 22 participants. The primary wearable signature was restricted to bilateral elbow and thigh sensors and should therefore be interpreted as a limb-focused representation of turning rather than as a comprehensive whole-body turning signature. Moreover, the extensive overlap between training sets and variation in fold-specific PCA loadings limit the interpretation of selection frequencies as evidence of reproducibility. This segment restriction was an exploratory analytic choice and was not independently validated before the present analysis. In future studies, researchers should prespecify sensor configurations, compare limb, axial, and whole-body signatures, and include external validation in larger independent cohorts. Finally, the ΔUPDRS Part III ≥ 1.5-point threshold was derived using H&Y worsening in the same 23 participants used for prognostic evaluation and therefore represents a cohort-specific, data-derived endpoint rather than an established clinical progression threshold. Training-fold preprocessing and feature selection do not independently validate this outcome definition. In the sensitivity analysis using H&Y worsening directly, adding the turning signature to the BL clinical model did not demonstrate incremental discrimination (AUC, 0.921 vs. 0.929; ΔAUC, 0.008; 95% CI, −0.032 to 0.063). Although this analysis avoided applying the 1.5-point threshold, it used the same participants and related motor outcomes and does not constitute independent validation. Further validation in larger prospective cohorts is needed to determine whether longitudinal changes in standardized turning measures provide complementary functional information for characterizing motor progression in PD alongside conventional clinical and imaging-based assessments.

## 5. Conclusions

In this exploratory longitudinal study, BL wearable-derived limb-focused turning movement-dynamics signatures were associated with worsening in UPDRS Part III. They demonstrated higher observed discrimination of motor progression than the examined BL DAT measures. These findings suggest that dynamic BL turning performance captures functional aspects of disease progression not fully reflected by a single BL measure of dopaminergic integrity. Rather than serving as an alternative to DAT imaging, wearable-derived turning measures may provide complementary information for characterizing motor progression in PD. Larger prospective studies are needed to establish the reproducibility and clinical relevance of these findings. In addition, motor progression was defined using a cohort-specific, H&Y-anchored exploratory threshold of ΔUPDRS Part III ≥ 1.5 points derived within the same cohort; this value should not be interpreted as a clinically established progression threshold. External validation in larger and independent cohorts is required before the progression definition or the turning-derived signature can be applied clinically.

## Figures and Tables

**Table 1:** Demographic, clinical, and ^18^F-FP-CIT PET characteristics at baseline and final follow-up of individuals with PD.

Characteristics	Baseline	Final Follow-Up	Change
Demographic characteristics			
Sex, men/women, *n*	12/11		-
Age (years)	69.26 ± 5.04	71.04 ± 5.07	+1.78 ± 0.42
Height (cm)	158.99 ± 7.07	158.40 ± 7.25	−0.58 ± 0.67
Weight (kg)	61.02 ± 9.70	60.85 ± 9.81	−0.17 ± 2.35
Body mass index (kg/m^2^)	24.00 ± 2.43	24.12 ± 2.41	+0.12 ± 0.94
Symptom duration (years)	5.07 ± 2.20	6.85 ± 2.30	+1.78 ± 0.24
Treatment duration (years)	4.24 ± 2.24	6.02 ± 2.34	+1.78 ± 0.24
L-dopa equivalent dose (mg/day)	417.49 ± 205.87	479.78 ± 271.93	+62.29 ± 233.10
K-MMSE (scores)	27.39 ± 2.27	27.87 ± 2.14	+0.48 ± 1.83
MoCA-K (scores)	25.35 ± 3.59	25.52 ± 3.87	+0.17 ± 2.95
UPDRS Total (scores)	48.43 ± 19.45	45.59 ± 12.77	−2.85 ± 13.61
UPDRS Part III (scores)	27.57 ± 14.11	28.41 ± 8.46	+0.85 ± 9.13
H&Y Stage, *n* (1/2/3)	7/14/2	0/20/3	Worsened 9/stable 13/improved 1
Regional ^18^F-FP-CIT SUVR			
Right caudate	4.07 ± 0.79	-	-
Left caudate	4.00 ± 0.71	-	-
Right putamen	3.08 ± 0.77	-	-
Left putamen	2.86 ± 0.54	-	-
Right striatum	3.64 ± 0.76	-	-
Left striatum	3.51 ± 0.63	-	-
Caudate asymmetry	7.20 ± 6.09	-	-
Putamen asymmetry	14.56 ± 9.48	-	-
Striatum asymmetry	9.77 ± 7.06	-	-

Continuous variables are presented as mean ± standard deviation, and categorical variables as number. For continuous variables assessed at both time points, change was calculated as the final follow-up value minus the baseline value. Accordingly, positive values indicate an increase, whereas negative values indicate a decrease from baseline to the final follow-up. A dash (-) indicates not assessed or not applicable. PET-derived measures were obtained only at baseline, and no change score was calculated for sex. For a change in the H&Y stage, at the final follow-up, no participants were classified as H&Y stage 1, 20 were classified as stage 2, and 3 were classified as stage 3. At the individual level, the H&Y stage worsened in 9 participants (stage 1 to 2, *n* = 7; stage 2 to 3, *n* = 2), remained unchanged in 13 (stage 2 to 2, *n* = 12; stage 3 to 3, *n* = 1), and improved in 1 (stage 3 to 2, *n* = 1). PD: Parkinson’s disease, L-dopa: levodopa; K-MMSE, Korean Mini-Mental State Examination; MoCA-K, Korean version of the Montreal Cognitive Assessment; UPDRS: Unified Parkinson’s Disease Rating Scale; H&Y: Hoehn and Yahr; SUVR: Standardized Uptake Value Ratio.

**Table 2:** Comparison of internally cross-validated discrimination of models for study-defined motor progression.

Model	AUC (95% CI)	Accuracy	Balanced Accuracy	F1 Score	Sensitivity	Specificity	PPV	NPV
BL UPDRS Part III	0.947 (0.818–1.000)	0.913	0.913	0.917	0.917	0.909	0.917	0.909
BL H&Y stage	0.659 (0.394–0.879)	0.783	0.792	0.737	0.583	1.000	1.000	0.688
BL UPDRS Part III + H&Y	0.962 (0.864–1.000)	0.826	0.830	0.818	0.750	0.909	0.900	0.769
Regional DAT signature	0.500 (0.250–0.758)	0.522	0.519	0.560	0.583	0.455	0.538	0.500
DAT burden	0.470 (0.223–0.725)	0.565	0.568	0.545	0.500	0.636	0.600	0.538
DAT asymmetry	0.462 (0.222–0.745)	0.609	0.602	0.667	0.750	0.455	0.600	0.625
Turning signature	0.750 (0.515–0.955)	0.652	0.648	0.692	0.750	0.545	0.643	0.667
Clinical model + turning signature	0.871 (0.674–1.000)	0.870	0.871	0.870	0.833	0.909	0.909	0.833

AUCs were calculated from pooled OOF predicted probabilities generated using LOOCV. DAT burden and DAT asymmetry denote separate univariable logistic regression models using the respective predefined composite score as the sole predictor. All threshold-dependent classification measures were calculated using an OOF predicted-probability threshold of ≥0.50. The clinical model included BL UPDRS Part III and H&Y stage. The combined model included BL UPDRS Part III, H&Y stage, and the fold-specific turning signature. BL, baseline; H&Y, Hoehn and Yahr; AUC, area under the receiver operating characteristic curve; DAT, dopamine transporter; LOOCV, leave-one-out cross-validation; OOF, out-of-fold; UPDRS, Unified Parkinson’s Disease Rating Scale; CI, confidence interval; PPV, positive predictive value; NPV, negative predictive value.

## Data Availability

The data presented in this study are available on request from the corresponding author due to ethical and privacy considerations related to the protection of participant confidentiality. Requests for access to the data will be considered on a case-by-case basis, subject to applicable ethical and institutional requirements.

## Supplementary Materials

The following supporting information can be downloaded at https://www.mdpi.com/article/10.3390/s26206610/s1, Table S1: Participant-specific assessment schedule; Table S2: Demographic, clinical, and ^18^F-FP-CIT PET characteristics for the overall and by motor progression status; Table S3: Wearable gait feature definitions; Table S4: Explained variance for the main turning-derived signature PCA; Table S5: Principal component loadings for the main turning-derived signature PCA; Table S6: Selection frequencies of baseline limb-focused turning movement-dynamics features across fully nested leave-one-out cross-validation folds.

## Abbreviations

The following abbreviations are used in this manuscript:

PD	Parkisnson’s disease
PET	Positron emission tomography
K-MMSE	Mini-Mental State Examination
CT	Computed Tomography
SUVR	Standardized Uptake Value Ratio
RMS	root mean square
PCA	principal component analysis
LOOCV	leave-one-out cross-validation
DAT	Dopamine transporter
AUC	area under the ROC curve
ΔUPDRS Part III	Δ Unified Parkinson’s Disease Rating Scale Part III
