# Supplementary material for: Baseline Wearable Turning-Derived Signature and Dopamine Transporter-Derived Measures in Relation to Subsequent Motor Progression in Parkinson’s Disease: An Exploratory Longitudinal Study
Source: Sensors (Basel). 2026 Oct 19;26(20):6610. doi: 10.3390/s26206610 (PMC13708933; doi:10.3390/s26206610)
Supplement: Supplementary file 1 [file sensors-26-06610-s001.zip › sensors-4582272-supplementary.pdf]

Table S1. Participant-specific assessment schedule

All 23 participants completed three assessments. Follow-up intervals are calculated from the participant's baseline assessment date.

Study ID	Baseline (BL) assessment date	First follow-up (T1) date	BL-to-T1 interval (months)	Final follow-up (T2) date	BL-to-final interval (months)
P01	2022-11-10	2023-11-30	12.65	2024-12-06	24.87
P02	2022-11-24	2023-11-20	11.86	2024-12-02	24.28
P03	2022-11-24	2023-11-27	12.09	2024-12-02	24.28
P04	2022-11-28	2023-11-24	11.86	2024-12-09	24.38
P05	2022-12-05	2023-11-27	11.73	2024-12-13	24.28
P06	2022-12-12	2023-11-30	11.60	2024-12-09	23.92
P07	2022-12-14	2023-11-23	11.30	2024-12-02	23.62
P08	2022-12-23	2023-11-27	11.14	2024-12-06	23.46
P09	2022-12-23	2023-11-23	11.01	2024-12-06	23.46
P10	2023-01-03	2023-11-27	10.78	2024-12-06	23.10
P11	2023-02-09	2023-11-29	9.63	2024-12-02	21.75
P12	2023-02-10	2023-11-22	9.36	2024-12-13	22.08
P13	2023-02-13	2023-11-22	9.26	2024-12-06	21.75
P14	2023-03-17	2023-11-22	8.21	2024-12-02	20.57
P15	2023-03-24	2023-11-22	7.98	2024-12-02	20.34
P16	2023-03-24	2023-11-22	7.98	2024-12-16	20.80
P17	2023-03-24	2023-11-29	8.21	2024-12-02	20.34
P18	2023-03-29	2023-11-22	7.82	2024-12-06	20.30
P19	2023-04-12	2023-11-22	7.36	2024-12-05	19.81
P20	2023-04-12	2023-11-22	7.36	2024-12-05	19.81
P21	2023-04-14	2023-11-22	7.29	2024-12-05	19.75
P22	2023-04-26	2023-11-22	6.90	2024-12-09	19.48
P23	2023-04-26	2023-11-22	6.90	2024-12-02	19.25

Notes: T1 denotes the first follow-up and T2 the final follow-up. Intervals in months were calculated as elapsed days divided by 30.4375. Dates are reported for the final analytic cohort only; Study IDs are publication-facing aliases.

Table S2. Demographic, clinical, and ^{18}F-FP-CIT PET characteristics for the overall and by motor progression status

Values are mean ± SD unless stated otherwise. For every continuous longitudinal variable, Change = final follow-up − BL. This table is descriptive; no p values are reported.

Characteristics			All participants			Progressor (n = 12)			Non-progressor (n = 11)			Difference in mean change (P – NP)
Domain	Characteristic	Unit / statistic	BL	Final follow-up	Change	BL	Final follow-up	Change	BL	Final follow-up	Change	
Study timeline	BL-to-final follow-up interval	months	—	21.99 ± 1.93	—	—	21.82 ± 2.01	—	—	22.16 ± 1.92	—	—
Demographic	Age	years	69.26 ± 5.04	71.04 ± 5.07	1.78 ± 0.42	67.83 ± 4.82	69.67 ± 4.77	1.83 ± 0.39	70.82 ± 5.02	72.55 ± 5.16	1.73 ± 0.47	0.11
	Height	cm	158.99 ± 7.07	158.40 ± 7.25	-0.58 ± 0.67	156.84 ± 5.61	156.32 ± 5.78	-0.53 ± 0.76	161.33 ± 7.99	160.68 ± 8.25	-0.65 ± 0.58	0.12
	Weight	kg	61.02 ± 9.70	60.85 ± 9.81	-0.17 ± 2.35	56.67 ± 8.36	56.95 ± 8.39	0.28 ± 1.83	65.76 ± 9.08	65.11 ± 9.81	-0.65 ± 2.81	0.94
	Body mass index	kg/m^2	24.00 ± 2.43	24.12 ± 2.41	0.12 ± 0.94	22.95 ± 2.58	23.23 ± 2.61	0.28 ± 0.81	25.15 ± 1.71	25.08 ± 1.83	-0.06 ± 1.08	0.35
Disease history	Symptom duration	years	5.07 ± 2.20	6.85 ± 2.30	1.78 ± 0.24	4.98 ± 2.41	6.75 ± 2.50	1.77 ± 0.25	5.17 ± 2.07	6.95 ± 2.17	1.78 ± 0.24	-0.01
	Treatment duration	years	4.24 ± 2.24	6.02 ± 2.34	1.78 ± 0.24	4.12 ± 2.16	5.89 ± 2.23	1.77 ± 0.25	4.37 ± 2.43	6.15 ± 2.55	1.78 ± 0.24	-0.01
Medication	Levodopa-equivalent daily dose	mg/day	417.49 ± 205.87	479.78 ± 271.93	62.29 ± 233.10	378.85 ± 230.02	480.42 ± 287.05	101.56 ± 141.72	459.64 ± 176.90	479.09 ± 268.40	19.45 ± 305.91	82.11
Cognition/mood	K-MMSE	score	27.39 ± 2.27	27.87 ± 2.14	0.48 ± 1.83	27.75 ± 2.22	28.42 ± 1.73	0.67 ± 2.10	27.00 ± 2.37	27.27 ± 2.45	0.27 ± 1.56	0.39
	MoCA-K	score	25.35 ± 3.59	25.52 ± 3.87	0.17 ± 2.95	26.25 ± 2.67	26.17 ± 3.69	-0.08 ± 3.65	24.36 ± 4.30	24.82 ± 4.12	0.45 ± 2.07	-0.54
	Beck Depression Inventory	score	12.96 ± 9.02	12.61 ± 8.77	-0.35 ± 10.90	14.75 ± 8.53	14.08 ± 8.93	-0.67 ± 10.08	11.00 ± 9.54	11.00 ± 8.72	0.00 ± 12.22	-0.67
	Beck Anxiety Inventory	score	28.91 ± 6.79	11.35 ± 8.46	-17.57 ± 8.92	28.42 ± 6.69	10.75 ± 8.75	-17.67 ± 10.18	29.45 ± 7.17	12.00 ± 8.51	-17.45 ± 7.81	-0.21
Motor/clinical	Hoehn and Yahr stage	stage	1.78 ± 0.60	2.13 ± 0.34	0.35 ± 0.57	1.42 ± 0.51	2.17 ± 0.39	0.75 ± 0.45	2.18 ± 0.40	2.09 ± 0.30	-0.09 ± 0.30	0.84
	UPDRS total	score	48.43 ± 19.45	45.59 ± 12.77	-2.85 ± 13.61	35.42 ± 12.31	42.17 ± 15.54	6.75 ± 7.69	62.64 ± 15.51	49.32 ± 7.98	-13.32 ± 10.56	20.07
	UPDRS Part I	score	9.39 ± 5.69	8.48 ± 5.38	-0.91 ± 4.08	8.67 ± 6.36	9.00 ± 6.59	0.33 ± 4.42	10.18 ± 5.06	7.91 ± 3.88	-2.27 ± 3.35	2.61
	UPDRS Part II	score	10.39 ± 5.51	8.30 ± 4.04	-2.09 ± 4.35	9.08 ± 4.76	7.67 ± 5.09	-1.42 ± 3.23	11.82 ± 6.13	9.00 ± 2.53	-2.82 ± 5.38	1.40
	UPDRS Part III	score	27.57 ± 14.11	28.41 ± 8.46	0.85 ± 9.13	17.50 ± 6.24	24.75 ± 6.82	7.25 ± 6.13	38.55 ± 11.86	32.41 ± 8.51	-6.14 ± 6.27	13.39
	UPDRS Part IV	score	1.09 ± 2.04	0.39 ± 1.88	-0.70 ± 2.93	0.17 ± 0.58	0.75 ± 2.60	0.58 ± 2.71	2.09 ± 2.59	0.00 ± 0.00	-2.09 ± 2.59	2.67
	Fall status (source coding)	code	1.87 ± 0.34	1.52 ± 0.51	-0.35 ± 0.49	1.83 ± 0.39	1.58 ± 0.51	-0.25 ± 0.45	1.91 ± 0.30	1.45 ± 0.52	-0.45 ± 0.52	0.20
	Korean Falls Efficacy Scale	score	20.83 ± 9.56	24.96 ± 10.19	4.13 ± 9.20	19.75 ± 8.34	23.17 ± 10.35	3.42 ± 8.26	22.00 ± 11.03	26.91 ± 10.12	4.91 ± 10.48	-1.49
	New Freezing of Gait Questionnaire	score	1.13 ± 4.45	2.09 ± 6.01	0.96 ± 3.89	2.17 ± 6.10	2.67 ± 7.10	0.50 ± 2.94	0.00 ± 0.00	1.45 ± 4.82	1.45 ± 4.82	-0.95
Patient-reported	PDQ-39	score	28.65 ± 15.53	30.96 ± 29.37	2.30 ± 24.15	28.25 ± 16.58	36.17 ± 33.73	7.92 ± 26.10	29.09 ± 15.09	25.27 ± 24.05	-3.82 ± 21.32	11.73
Physical performance	Left grip strength	source unit	25.79 ± 7.49	26.74 ± 8.55	0.95 ± 3.08	25.18 ± 7.21	26.53 ± 9.24	1.34 ± 3.10	26.45 ± 8.08	26.98 ± 8.18	0.53 ± 3.15	0.81
	Right grip strength	source unit	26.63 ± 7.74	26.83 ± 8.18	0.20 ± 3.56	25.24 ± 6.48	25.54 ± 7.42	0.30 ± 3.91	28.14 ± 9.00	28.23 ± 9.08	0.09 ± 3.32	0.21
	Five-times sit-to-stand	s	8.11 ± 2.89	7.70 ± 2.02	-0.40 ± 3.58	7.69 ± 2.11	6.84 ± 1.03	-0.85 ± 2.20	8.56 ± 3.61	8.65 ± 2.44	0.08 ± 4.73	-0.93
	Six-minute walk test	m	432.74 ± 103.73	424.49 ± 71.29	-8.25 ± 97.59	470.28 ± 104.43	462.82 ± 39.26	-7.46 ± 111.71	391.79 ± 90.23	382.68 ± 76.15	-9.11 ± 84.98	1.65
	Mini-BESTest	score	23.78 ± 2.97	25.83 ± 1.95	2.04 ± 3.17	24.08 ± 3.09	26.67 ± 1.61	2.58 ± 3.58	23.45 ± 2.94	24.91 ± 1.92	1.45 ± 2.70	1.13
	FOOD (source variable)	source unit	65.65 ± 17.81	69.09 ± 9.67	3.43 ± 14.43	63.33 ± 22.78	67.42 ± 9.92	4.08 ± 18.73	68.18 ± 10.64	70.91 ± 9.51	2.73 ± 8.45	1.36
Physical activity	Physical activity	source unit	2603.35 ± 2488.99	2451.15 ± 2259.13	-152.20 ± 2999.42	2954.75 ± 2777.61	3379.50 ± 2608.02	424.75 ± 3255.21	2220.00 ± 2198.34	1438.41 ± 1258.45	-781.59 ± 2702.06	1206.34
Demographic	Sex: Men	n (%)	12 (52.2%)	—	—	5 (41.7%)	—	—	7 (63.6%)	—	—	—
	Sex: Women	n (%)	11 (47.8%)	—	—	7 (58.3%)	—	—	4 (36.4%)	—	—	—
	Dominant/affected side (source coding): Code 1	n (%)	5 (21.7%)	—	—	3 (25.0%)	—	—	2 (18.2%)	—	—	—
	Dominant/affected side (source coding): Code 2	n (%)	12 (52.2%)	—	—	7 (58.3%)	—	—	5 (45.5%)	—	—	—
	Dominant/affected side (source coding): Code 3	n (%)	6 (26.1%)	—	—	2 (16.7%)	—	—	4 (36.4%)	—	—	—
Motor/clinical	H&Y trajectory: worsened	n (%)	—	—	9 (39.1%)	—	—	9 (75.0%)	—	—	0 (0.0%)	—
	H&Y trajectory: unchanged	n (%)	—	—	13 (56.5%)	—	—	3 (25.0%)	—	—	10 (90.9%)	—
	H&Y trajectory: improved	n (%)	—	—	1 (4.3%)	—	—	0 (0.0%)	—	—	1 (9.1%)	—
Baseline ^{18}F-FP-CIT PET	Right caudate uptake	source unit	4.065 ± 0.787	—	—	4.092 ± 0.877	—	—	4.036 ± 0.717	—	—	—
	Left caudate uptake	source unit	4.004 ± 0.710	—	—	4.100 ± 0.782	—	—	3.900 ± 0.643	—	—	—
	Right putamen uptake	source unit	3.083 ± 0.766	—	—	3.175 ± 0.867	—	—	2.982 ± 0.665	—	—	—
	Left putamen uptake	source unit	2.861 ± 0.544	—	—	2.992 ± 0.573	—	—	2.718 ± 0.498	—	—	—
	Right striatal uptake	source unit	3.639 ± 0.761	—	—	3.683 ± 0.861	—	—	3.591 ± 0.674	—	—	—
	Left striatal uptake	source unit	3.509 ± 0.627	—	—	3.617 ± 0.685	—	—	3.391 ± 0.565	—	—	—
	Caudate asymmetry index	asymmetry index	7.196 ± 6.092	—	—	5.858 ± 6.001	—	—	8.655 ± 6.128	—	—	—
	Putamen asymmetry index	asymmetry index	14.557 ± 9.479	—	—	14.317 ± 7.876	—	—	14.818 ± 11.371	—	—	—
	Striatal asymmetry index	asymmetry index	9.770 ± 7.056	—	—	8.692 ± 6.612	—	—	10.945 ± 7.650	—	—	—
	Left putamen-to-caudate ratio	ratio	0.722 ± 0.074	—	—	0.733 ± 0.089	—	—	0.709 ± 0.054	—	—	—
	Right putamen-to-caudate ratio	ratio	0.752 ± 0.085	—	—	0.775 ± 0.087	—	—	0.727 ± 0.079	—	—	—
	Right functional striatal volume	source unit	4.617 ± 1.087	—	—	4.450 ± 0.984	—	—	4.800 ± 1.209	—	—	—
	Right functional striatal activity	source unit	37.857 ± 17.097	—	—	37.508 ± 17.909	—	—	38.236 ± 17.029	—	—	—

	Left functional striatal volume	source unit	4.370 ± 1.130	—	—	4.517 ± 0.953	—	—	4.209 ± 1.325	—	—	—
	Left functional striatal activity	source unit	35.409 ± 16.652	—	—	37.150 ± 16.430	—	—	33.509 ± 17.479	—	—	—
	Right anterior/posterior putamen ratio	ratio	1.778 ± 0.431	—	—	1.767 ± 0.442	—	—	1.791 ± 0.439	—	—	—
	Left anterior/posterior putamen ratio	ratio	1.874 ± 0.435	—	—	1.942 ± 0.538	—	—	1.800 ± 0.293	—	—	—
	Occipital reference uptake	source unit	1.583 ± 0.531	—	—	1.600 ± 0.569	—	—	1.564 ± 0.514	—	—	—
	Cerebellar uptake	source unit	1.083 ± 0.337	—	—	1.100 ± 0.379	—	—	1.064 ± 0.301	—	—	—
**Analysis-derived markers**	DAT burden score	z-score	0.000 ± 0.958	—	—	-0.151 ± 1.070	—	—	0.165 ± 0.838	—	—	—
	DAT asymmetry score	z-score	0.000 ± 0.978	—	—	-0.136 ± 0.905	—	—	0.148 ± 1.076	—	—	—
	Risk-aligned OOF turning signature (K=5)	OOF score	-0.017 ± 1.584	—	—	-0.121 ± 1.949	—	—	0.095 ± 1.146	—	—	—
	OOF predicted progression probability (K=5)	probability	0.492 ± 0.215	—	—	0.563 ± 0.200	—	—	0.415 ± 0.214	—	—	—

Notes: Motor progression was defined using the cohort-specific H&Y-anchored threshold of ΔUPDRS Part III $\geq$ 1.5 points. For all continuous longitudinal variables, Change was calculated as final follow-up minus BL; positive and negative values therefore indicate increases and decreases, respectively. PET and OOF-derived measures are BL-only. H&Y stage is ordinal; mean ± SD is shown only to retain the table-wide descriptive convention, with categorical trajectories reported separately.

Supplementary Table S3. Wearable gait feature definitions

Feature family	Variable	Trial-level calculation in supplied code	Units / qualification	Interpretation / implementation qualification
Movement magnitude	Acceleration RMS (rms_acc)	$\mathrm{sqrt}((1/N) \times \Sigma t\ [ax(t)^2 + ay(t)^2 + az(t)^2])$	Input acceleration units	RMS of triaxial acceleration magnitude. No gravity removal or time-series centering is implemented in these scripts.
Movement magnitude	Angular-velocity RMS (rms_gyr)	$\mathrm{sqrt}((1/N) \times \Sigma t\ [\omega x(t)^2 + \omega y(t)^2 + \omega z(t)^2])$	Input angular-velocity units	RMS of triaxial angular-velocity magnitude. RMS measures signal magnitude.
Signal irregularity	First-axis acceleration sample entropy (saen_acc)	$-\ln(Aacc/Bacc)$, using $ax(t)$; $m = 2$, $\tau = 1$; $r = 0.2 \times \mathrm{SD}(\mathrm{vec}([a, \omega]))$.	Dimensionless	SampEn.m uses linear indexing on the $N \times 3$ input, so templates use the first axis only for $N > 3$. The tolerance pools all six input
Signal irregularity	First-axis angular-velocity sample entropy (saen_gyr)	$-\ln(Agyr/Bgyr)$, using $\omega x(t)$; $m = 2$, $\tau = 1$; the same pooled r as saen_acc.	Dimensionless	The first angular-velocity axis enters the templates. Other axes and acceleration affect the pooled tolerance. This is not triaxial or vector-magnitude entropy.
Movement magnitude	Mean acceleration magnitude (mean_acc)	$\mu a = (1/N) \times \Sigma t\ \mathrm{sqrt}[ax(t)^2 + ay(t)^2 + az(t)^2]$	Input acceleration units	Time-averaged Euclidean magnitude. μa also supplies both finite-difference denominators.
Movement magnitude	Peak acceleration magnitude (max_acc)	$\max\_t\ \mathrm{sqrt}[ax(t)^2 + ay(t)^2 + az(t)^2]$	Input acceleration units	Peak Euclidean magnitude.
Movement magnitude	Mean angular-velocity magnitude (mean_gyr)	$(1/N) \times \Sigma t\ \mathrm{sqrt}[\omega x(t)^2 + \omega y(t)^2 + \omega z(t)^2]$	Input angular-velocity units	Time-averaged Euclidean magnitude.
Movement magnitude	Peak angular-velocity magnitude (max_gyr)	$\max\_t\ \mathrm{sqrt}[\omega x(t)^2 + \omega y(t)^2 + \omega z(t)^2]$	Input angular-velocity units	Peak Euclidean magnitude.
Finite differences	Acceleration first-difference index (stored as max_jerk)	$[(1/3) \times \Sigma q\ \max\_t\ \lvert aq(t+1) - aq(t)\rvert] / \mu a^2$, $q \in \{x,y,z\}$.	$(\text{acceleration unit})^{-1}$	Mean of axis-specific peak absolute differences, divided twice by μa. No Δt scaling. This is not physical maximal jerk in m/s^3.
Finite differences	Angular-velocity first-difference index (stored as max_angvel_jerk)	$[(1/3) \times \Sigma q\ \max\_t\ \lvert \omega q(t+1) - \omega q(t)\rvert] / \mu a^2$, $q \in \{x,y,z\}$.	$(\text{angular-velocity unit}) / (\text{acceleration unit})^2$	Active code uses a first difference and acceleration-based normalization twice. The earlier second-difference lines are commented out. No Δt scaling.

Notation: a and ω are N × 3 matrices. vec stacks columns. μa is mean_acc. SD uses MATLAB std with its default sample denominator (6N − 1). Units are not converted in these scripts; confirm the exported CSV units before assigning
Trial aggregation: calculate each feature separately for each trial, then use the arithmetic mean of the three trial-level values within participant, visit, task condition and sensor. This step was confirmed by the author but is not
Entropy matching: use N − 2 templates at both lengths 2 and 3. A/B count unordered distinct template pairs with Chebyshev distance strictly < r. Natural logarithm; self-matches excluded. No pseudocount or Inf/NaN handling is
The primary turning feature family combines two triaxial RMS features with two first-axis entropy features in the supplied implementation. The shared tolerance combines acceleration and angular velocity without channel-wise scaling,
The two stored jerk columns are normalized first-difference indices, not time derivatives. The denominator is μa^2 for both. If physical jerk measures are required, a revised definition and recalculation would constitute an analysis
Domain_Walk.m retains rows from the first column-1 value ≥ 100 to the file end; Domain_Turn.m uses ≥ 150. These are initial-trimming rules, not detected movement onset/offset. Column-1 meaning and recording-stop procedure

Table S4. Explained variance for the main turning-derived signature PCA

PCA was fitted separately at BL for each turning-condition × body-segment combination using four turning-derived features. Two PCs were retained for all 16 combinations.

Turning condition	Body segment	Task code	PCA domain	Original features, n	Retained PCs, n	PC1 variance (%)	PC2 variance (%)	Cumulative variance (%)	Retention rule
Fast left turn	Left elbow	XFASTL	XFASTL_LELB_variability	4	2	74.99	16.81	91.81	Cumulative variance ≥80%; maximum 2 PCs
Fast left turn	Left thigh	XFASTL	XFASTL_LTHI_variability	4	2	78.67	12.18	90.85	Cumulative variance ≥80%; maximum 2 PCs
Fast left turn	Right elbow	XFASTL	XFASTL_RELB_variability	4	2	64.36	29.54	93.90	Cumulative variance ≥80%; maximum 2 PCs
Fast left turn	Right thigh	XFASTL	XFASTL_RTHI_variability	4	2	75.50	15.36	90.86	Cumulative variance ≥80%; maximum 2 PCs
Fast right turn	Left elbow	XFASTR	XFASTR_LELB_variability	4	2	71.26	18.97	90.23	Cumulative variance ≥80%; maximum 2 PCs
Fast right turn	Left thigh	XFASTR	XFASTR_LTHI_variability	4	2	78.82	14.57	93.40	Cumulative variance ≥80%; maximum 2 PCs
Fast right turn	Right elbow	XFASTR	XFASTR_RELB_variability	4	2	69.15	24.85	94.01	Cumulative variance ≥80%; maximum 2 PCs
Fast right turn	Right thigh	XFASTR	XFASTR_RTHI_variability	4	2	75.57	15.11	90.68	Cumulative variance ≥80%; maximum 2 PCs
Preferred-speed left turn	Left elbow	XPREFL	XPREFL_LELB_variability	4	2	71.49	19.58	91.07	Cumulative variance ≥80%; maximum 2 PCs
Preferred-speed left turn	Left thigh	XPREFL	XPREFL_LTHI_variability	4	2	77.51	14.26	91.77	Cumulative variance ≥80%; maximum 2 PCs
Preferred-speed left turn	Right elbow	XPREFL	XPREFL_RELB_variability	4	2	66.65	25.57	92.21	Cumulative variance ≥80%; maximum 2 PCs
Preferred-speed left turn	Right thigh	XPREFL	XPREFL_RTHI_variability	4	2	75.02	14.61	89.63	Cumulative variance ≥80%; maximum 2 PCs
Preferred-speed right turn	Left elbow	XPREFR	XPREFR_LELB_variability	4	2	70.99	20.10	91.09	Cumulative variance ≥80%; maximum 2 PCs
Preferred-speed right turn	Left thigh	XPREFR	XPREFR_LTHI_variability	4	2	77.59	14.38	91.97	Cumulative variance ≥80%; maximum 2 PCs
Preferred-speed right turn	Right elbow	XPREFR	XPREFR_RELB_variability	4	2	67.93	25.35	93.29	Cumulative variance ≥80%; maximum 2 PCs
Preferred-speed right turn	Right thigh	XPREFR	XPREFR_RTHI_variability	4	2	73.60	16.85	90.45	Cumulative variance ≥80%; maximum 2 PCs

	PC1 variance (%)	PC2 variance (%)	Cumulative variance (%)	
**Across-combination mean**	**73.070**	**18.631**	**91.701**	**Descriptive summary**
**Across-combination minimum**	**64.363**	**12.176**	**89.634**	**Reported range**
**Across-combination maximum**	**78.824**	**29.537**	**94.007**	**Reported range**

Table S5. Principal component loadings for the main turning-derived signature PCA

Full-precision loadings are shown in long format. Absolute loading is included for ranking; component signs are arbitrary and aligned with the manuscript-facing PCA table.

Turning condition	Body segment	Task code	PCA domain	PC	Explained variance (%)	Source variable	Sensor-derived metric	Loading	Absolute loading
Fast left turn	Left elbow	XFASTL	XFASTL_LELB_variability	PC1	74.993	XFASTL_rms_gyr_LELB	RMS angular velocity	-0.435	0.435
Fast left turn	Left elbow	XFASTL	XFASTL_LELB_variability	PC1	74.993	XFASTL_rms_acc_LELB	RMS acceleration	-0.548	0.548
Fast left turn	Left elbow	XFASTL	XFASTL_LELB_variability	PC1	74.993	XFASTL_saen_acc_LELB	Sample entropy, acceleration	0.510	0.510
Fast left turn	Left elbow	XFASTL	XFASTL_LELB_variability	PC1	74.993	XFASTL_saen_gyr_LELB	Sample entropy, angular velocity	-0.501	0.501
Fast left turn	Left elbow	XFASTL	XFASTL_LELB_variability	PC2	16.813	XFASTL_rms_gyr_LELB	RMS angular velocity	-0.783	0.783
Fast left turn	Left elbow	XFASTL	XFASTL_LELB_variability	PC2	16.813	XFASTL_rms_acc_LELB	RMS acceleration	-0.152	0.152
Fast left turn	Left elbow	XFASTL	XFASTL_LELB_variability	PC2	16.813	XFASTL_saen_acc_LELB	Sample entropy, acceleration	-0.342	0.342
Fast left turn	Left elbow	XFASTL	XFASTL_LELB_variability	PC2	16.813	XFASTL_saen_gyr_LELB	Sample entropy, angular velocity	0.497	0.497
Fast left turn	Left thigh	XFASTL	XFASTL_LTHI_variability	PC1	78.672	XFASTL_rms_gyr_LTHI	RMS angular velocity	-0.470	0.470
Fast left turn	Left thigh	XFASTL	XFASTL_LTHI_variability	PC1	78.672	XFASTL_rms_acc_LTHI	RMS acceleration	-0.533	0.533
Fast left turn	Left thigh	XFASTL	XFASTL_LTHI_variability	PC1	78.672	XFASTL_saen_acc_LTHI	Sample entropy, acceleration	0.477	0.477
Fast left turn	Left thigh	XFASTL	XFASTL_LTHI_variability	PC1	78.672	XFASTL_saen_gyr_LTHI	Sample entropy, angular velocity	-0.517	0.517
Fast left turn	Left thigh	XFASTL	XFASTL_LTHI_variability	PC2	12.176	XFASTL_rms_gyr_LTHI	RMS angular velocity	-0.730	0.730
Fast left turn	Left thigh	XFASTL	XFASTL_LTHI_variability	PC2	12.176	XFASTL_rms_acc_LTHI	RMS acceleration	-0.098	0.098
Fast left turn	Left thigh	XFASTL	XFASTL_LTHI_variability	PC2	12.176	XFASTL_saen_acc_LTHI	Sample entropy, acceleration	-0.657	0.657
Fast left turn	Left thigh	XFASTL	XFASTL_LTHI_variability	PC2	12.176	XFASTL_saen_gyr_LTHI	Sample entropy, angular velocity	0.159	0.159
Fast left turn	Right elbow	XFASTL	XFASTL_RELB_variability	PC1	64.363	XFASTL_rms_gyr_RELB	RMS angular velocity	-0.526	0.526
Fast left turn	Right elbow	XFASTL	XFASTL_RELB_variability	PC1	64.363	XFASTL_rms_acc_RELB	RMS acceleration	-0.601	0.601
Fast left turn	Right elbow	XFASTL	XFASTL_RELB_variability	PC1	64.363	XFASTL_saen_acc_RELB	Sample entropy, acceleration	0.585	0.585
Fast left turn	Right elbow	XFASTL	XFASTL_RELB_variability	PC1	64.363	XFASTL_saen_gyr_RELB	Sample entropy, angular velocity	-0.138	0.138
Fast left turn	Right elbow	XFASTL	XFASTL_RELB_variability	PC2	29.537	XFASTL_rms_gyr_RELB	RMS angular velocity	-0.398	0.398
Fast left turn	Right elbow	XFASTL	XFASTL_RELB_variability	PC2	29.537	XFASTL_rms_acc_RELB	RMS acceleration	-0.084	0.084
Fast left turn	Right elbow	XFASTL	XFASTL_RELB_variability	PC2	29.537	XFASTL_saen_acc_RELB	Sample entropy, acceleration	-0.235	0.235
Fast left turn	Right elbow	XFASTL	XFASTL_RELB_variability	PC2	29.537	XFASTL_saen_gyr_RELB	Sample entropy, angular velocity	0.883	0.883
Fast left turn	Right thigh	XFASTL	XFASTL_RTHI_variability	PC1	75.505	XFASTL_rms_gyr_RTHI	RMS angular velocity	-0.457	0.457
Fast left turn	Right thigh	XFASTL	XFASTL_RTHI_variability	PC1	75.505	XFASTL_rms_acc_RTHI	RMS acceleration	-0.541	0.541
Fast left turn	Right thigh	XFASTL	XFASTL_RTHI_variability	PC1	75.505	XFASTL_saen_acc_RTHI	Sample entropy, acceleration	0.494	0.494
Fast left turn	Right thigh	XFASTL	XFASTL_RTHI_variability	PC1	75.505	XFASTL_saen_gyr_RTHI	Sample entropy, angular velocity	-0.504	0.504
Fast left turn	Right thigh	XFASTL	XFASTL_RTHI_variability	PC2	15.359	XFASTL_rms_gyr_RTHI	RMS angular velocity	-0.742	0.742
Fast left turn	Right thigh	XFASTL	XFASTL_RTHI_variability	PC2	15.359	XFASTL_rms_acc_RTHI	RMS acceleration	-0.206	0.206
Fast left turn	Right thigh	XFASTL	XFASTL_RTHI_variability	PC2	15.359	XFASTL_saen_acc_RTHI	Sample entropy, acceleration	-0.446	0.446
Fast left turn	Right thigh	XFASTL	XFASTL_RTHI_variability	PC2	15.359	XFASTL_saen_gyr_RTHI	Sample entropy, angular velocity	0.457	0.457
Fast right turn	Left elbow	XFASTR	XFASTR_LELB_variability	PC1	71.259	XFASTR_rms_gyr_LELB	RMS angular velocity	-0.445	0.445
Fast right turn	Left elbow	XFASTR	XFASTR_LELB_variability	PC1	71.259	XFASTR_rms_acc_LELB	RMS acceleration	-0.562	0.562
Fast right turn	Left elbow	XFASTR	XFASTR_LELB_variability	PC1	71.259	XFASTR_saen_acc_LELB	Sample entropy, acceleration	0.522	0.522
Fast right turn	Left elbow	XFASTR	XFASTR_LELB_variability	PC1	71.259	XFASTR_saen_gyr_LELB	Sample entropy, angular velocity	-0.462	0.462
Fast right turn	Left elbow	XFASTR	XFASTR_LELB_variability	PC2	18.973	XFASTR_rms_gyr_LELB	RMS angular velocity	-0.725	0.725
Fast right turn	Left elbow	XFASTR	XFASTR_LELB_variability	PC2	18.973	XFASTR_rms_acc_LELB	RMS acceleration	-0.154	0.154
Fast right turn	Left elbow	XFASTR	XFASTR_LELB_variability	PC2	18.973	XFASTR_saen_acc_LELB	Sample entropy, acceleration	-0.225	0.225
Fast right turn	Left elbow	XFASTR	XFASTR_LELB_variability	PC2	18.973	XFASTR_saen_gyr_LELB	Sample entropy, angular velocity	0.632	0.632
Fast right turn	Left thigh	XFASTR	XFASTR_LTHI_variability	PC1	78.824	XFASTR_rms_gyr_LTHI	RMS angular velocity	-0.453	0.453
Fast right turn	Left thigh	XFASTR	XFASTR_LTHI_variability	PC1	78.824	XFASTR_rms_acc_LTHI	RMS acceleration	-0.532	0.532
Fast right turn	Left thigh	XFASTR	XFASTR_LTHI_variability	PC1	78.824	XFASTR_saen_acc_LTHI	Sample entropy, acceleration	0.503	0.503
Fast right turn	Left thigh	XFASTR	XFASTR_LTHI_variability	PC1	78.824	XFASTR_saen_gyr_LTHI	Sample entropy, angular velocity	-0.508	0.508
Fast right turn	Left thigh	XFASTR	XFASTR_LTHI_variability	PC2	14.573	XFASTR_rms_gyr_LTHI	RMS angular velocity	-0.746	0.746
Fast right turn	Left thigh	XFASTR	XFASTR_LTHI_variability	PC2	14.573	XFASTR_rms_acc_LTHI	RMS acceleration	-0.212	0.212
Fast right turn	Left thigh	XFASTR	XFASTR_LTHI_variability	PC2	14.573	XFASTR_saen_acc_LTHI	Sample entropy, acceleration	-0.445	0.445
Fast right turn	Left thigh	XFASTR	XFASTR_LTHI_variability	PC2	14.573	XFASTR_saen_gyr_LTHI	Sample entropy, angular velocity	0.447	0.447
Fast right turn	Right elbow	XFASTR	XFASTR_RELB_variability	PC1	69.152	XFASTR_rms_gyr_RELB	RMS angular velocity	-0.486	0.486
Fast right turn	Right elbow	XFASTR	XFASTR_RELB_variability	PC1	69.152	XFASTR_rms_acc_RELB	RMS acceleration	-0.576	0.576
Fast right turn	Right elbow	XFASTR	XFASTR_RELB_variability	PC1	69.152	XFASTR_saen_acc_RELB	Sample entropy, acceleration	0.572	0.572
Fast right turn	Right elbow	XFASTR	XFASTR_RELB_variability	PC1	69.152	XFASTR_saen_gyr_RELB	Sample entropy, angular velocity	-0.324	0.324
Fast right turn	Right elbow	XFASTR	XFASTR_RELB_variability	PC2	24.855	XFASTR_rms_gyr_RELB	RMS angular velocity	-0.526	0.526
Fast right turn	Right elbow	XFASTR	XFASTR_RELB_variability	PC2	24.855	XFASTR_rms_acc_RELB	RMS acceleration	-0.158	0.158
Fast right turn	Right elbow	XFASTR	XFASTR_RELB_variability	PC2	24.855	XFASTR_saen_acc_RELB	Sample entropy, acceleration	-0.138	0.138
Fast right turn	Right elbow	XFASTR	XFASTR_RELB_variability	PC2	24.855	XFASTR_saen_gyr_RELB	Sample entropy, angular velocity	0.824	0.824
Fast right turn	Right thigh	XFASTR	XFASTR_RTHI_variability	PC1	75.569	XFASTR_rms_gyr_RTHI	RMS angular velocity	-0.460	0.460
Fast right turn	Right thigh	XFASTR	XFASTR_RTHI_variability	PC1	75.569	XFASTR_rms_acc_RTHI	RMS acceleration	-0.550	0.550
Fast right turn	Right thigh	XFASTR	XFASTR_RTHI_variability	PC1	75.569	XFASTR_saen_acc_RTHI	Sample entropy, acceleration	0.487	0.487
Fast right turn	Right thigh	XFASTR	XFASTR_RTHI_variability	PC1	75.569	XFASTR_saen_gyr_RTHI	Sample entropy, angular velocity	-0.499	0.499
Fast right turn	Right thigh	XFASTR	XFASTR_RTHI_variability	PC2	15.110	XFASTR_rms_gyr_RTHI	RMS angular velocity	-0.751	0.751
Fast right turn	Right thigh	XFASTR	XFASTR_RTHI_variability	PC2	15.110	XFASTR_rms_acc_RTHI	RMS acceleration	-0.178	0.178
Fast right turn	Right thigh	XFASTR	XFASTR_RTHI_variability	PC2	15.110	XFASTR_saen_acc_RTHI	Sample entropy, acceleration	-0.421	0.421
Fast right turn	Right thigh	XFASTR	XFASTR_RTHI_variability	PC2	15.110	XFASTR_saen_gyr_RTHI	Sample entropy, angular velocity	0.477	0.477
Preferred-speed left turn	Left elbow	XPREFL	XPREFL_LELB_variability	PC1	71.486	XPREFL_rms_gyr_LELB	RMS angular velocity	-0.413	0.413
Preferred-speed left turn	Left elbow	XPREFL	XPREFL_LELB_variability	PC1	71.486	XPREFL_rms_acc_LELB	RMS acceleration	-0.565	0.565
Preferred-speed left turn	Left elbow	XPREFL	XPREFL_LELB_variability	PC1	71.486	XPREFL_saen_acc_LELB	Sample entropy, acceleration	0.522	0.522
Preferred-speed left turn	Left elbow	XPREFL	XPREFL_LELB_variability	PC1	71.486	XPREFL_saen_gyr_LELB	Sample entropy, angular velocity	-0.488	0.488
Preferred-speed left turn	Left elbow	XPREFL	XPREFL_LELB_variability	PC2	19.584	XPREFL_rms_gyr_LELB	RMS angular velocity	-0.794	0.794
Preferred-speed left turn	Left elbow	XPREFL	XPREFL_LELB_variability	PC2	19.584	XPREFL_rms_acc_LELB	RMS acceleration	-0.102	0.102
Preferred-speed left turn	Left elbow	XPREFL	XPREFL_LELB_variability	PC2	19.584	XPREFL_saen_acc_LELB	Sample entropy, acceleration	-0.217	0.217
Preferred-speed left turn	Left elbow	XPREFL	XPREFL_LELB_variability	PC2	19.584	XPREFL_saen_gyr_LELB	Sample entropy, angular velocity	0.559	0.559
Preferred-speed left turn	Left thigh	XPREFL	XPREFL_LTHI_variability	PC1	77.515	XPREFL_rms_gyr_LTHI	RMS angular velocity	-0.446	0.446
Preferred-speed left turn	Left thigh	XPREFL	XPREFL_LTHI_variability	PC1	77.515	XPREFL_rms_acc_LTHI	RMS acceleration	-0.539	0.539
Preferred-speed left turn	Left thigh	XPREFL	XPREFL_LTHI_variability	PC1	77.515	XPREFL_saen_acc_LTHI	Sample entropy, acceleration	0.498	0.498
Preferred-speed left turn	Left thigh	XPREFL	XPREFL_LTHI_variability	PC1	77.515	XPREFL_saen_gyr_LTHI	Sample entropy, angular velocity	-0.513	0.513
Preferred-speed left turn	Left thigh	XPREFL	XPREFL_LTHI_variability	PC2	14.255	XPREFL_rms_gyr_LTHI	RMS angular velocity	-0.795	0.795
Preferred-speed left turn	Left thigh	XPREFL	XPREFL_LTHI_variability	PC2	14.255	XPREFL_rms_acc_LTHI	RMS acceleration	-0.128	0.128
Preferred-speed left turn	Left thigh	XPREFL	XPREFL_LTHI_variability	PC2	14.255	XPREFL_saen_acc_LTHI	Sample entropy, acceleration	-0.430	0.430
Preferred-speed left turn	Left thigh	XPREFL	XPREFL_LTHI_variability	PC2	14.255	XPREFL_saen_gyr_LTHI	Sample entropy, angular velocity	0.407	0.407
Preferred-speed left turn	Right elbow	XPREFL	XPREFL_RELB_variability	PC1	66.649	XPREFL_rms_gyr_RELB	RMS angular velocity	-0.484	0.484
Preferred-speed left turn	Right elbow	XPREFL	XPREFL_RELB_variability	PC1	66.649	XPREFL_rms_acc_RELB	RMS acceleration	-0.586	0.586
Preferred-speed left turn	Right elbow	XPREFL	XPREFL_RELB_variability	PC1	66.649	XPREFL_saen_acc_RELB	Sample entropy, acceleration	0.579	0.579
Preferred-speed left turn	Right elbow	XPREFL	XPREFL_RELB_variability	PC1	66.649	XPREFL_saen_gyr_RELB	Sample entropy, angular velocity	-0.295	0.295
Preferred-speed left turn	Right elbow	XPREFL	XPREFL_RELB_variability	PC2	25.565	XPREFL_rms_gyr_RELB	RMS angular velocity	-0.508	0.508
Preferred-speed left turn	Right elbow	XPREFL	XPREFL_RELB_variability	PC2	25.565	XPREFL_rms_acc_RELB	RMS acceleration	-0.135	0.135
Preferred-speed left turn	Right elbow	XPREFL	XPREFL_RELB_variability	PC2	25.565	XPREFL_saen_acc_RELB	Sample entropy, acceleration	-0.133	0.133
Preferred-speed left turn	Right elbow	XPREFL	XPREFL_RELB_variability	PC2	25.565	XPREFL_saen_gyr_RELB	Sample entropy, angular velocity	0.840	0.840
Preferred-speed left turn	Right thigh	XPREFL	XPREFL_RTHI_variability	PC1	75.023	XPREFL_rms_gyr_RTHI	RMS angular velocity	-0.459	0.459
Preferred-speed left turn	Right thigh	XPREFL	XPREFL_RTHI_variability	PC1	75.023	XPREFL_rms_acc_RTHI	RMS acceleration	-0.546	0.546
Preferred-speed left turn	Right thigh	XPREFL	XPREFL_RTHI_variability	PC1	75.023	XPREFL_saen_acc_RTHI	Sample entropy, acceleration	0.485	0.485
Preferred-speed left turn	Right thigh	XPREFL	XPREFL_RTHI_variability	PC1	75.023	XPREFL_saen_gyr_RTHI	Sample entropy, angular velocity	-0.505	0.505

Preferred-speed left turn	Right thigh	XPREFL	XPREFL_RTHI_variability	PC2	14.611	XPREFL_rms_gyr_RTHI	RMS angular velocity	-0.759	0.759
Preferred-speed left turn	Right thigh	XPREFL	XPREFL_RTHI_variability	PC2	14.611	XPREFL_rms_acc_RTHI	RMS acceleration	-0.164	0.164
Preferred-speed left turn	Right thigh	XPREFL	XPREFL_RTHI_variability	PC2	14.611	XPREFL_saen_acc_RTHI	Sample entropy, acceleration	-0.484	0.484
Preferred-speed left turn	Right thigh	XPREFL	XPREFL_RTHI_variability	PC2	14.611	XPREFL_saen_gyr_RTHI	Sample entropy, angular velocity	0.403	0.403
Preferred-speed right turn	Left elbow	XPREFR	XPREFR_LELB_variability	PC1	70.993	XPREFR_rms_gyr_LELB	RMS angular velocity	-0.435	0.435
Preferred-speed right turn	Left elbow	XPREFR	XPREFR_LELB_variability	PC1	70.993	XPREFR_rms_acc_LELB	RMS acceleration	-0.565	0.565
Preferred-speed right turn	Left elbow	XPREFR	XPREFR_LELB_variability	PC1	70.993	XPREFR_saen_acc_LELB	Sample entropy, acceleration	0.527	0.527
Preferred-speed right turn	Left elbow	XPREFR	XPREFR_LELB_variability	PC1	70.993	XPREFR_saen_gyr_LELB	Sample entropy, angular velocity	-0.463	0.463
Preferred-speed right turn	Left elbow	XPREFR	XPREFR_LELB_variability	PC2	20.100	XPREFR_rms_gyr_LELB	RMS angular velocity	-0.737	0.737
Preferred-speed right turn	Left elbow	XPREFR	XPREFR_LELB_variability	PC2	20.100	XPREFR_rms_acc_LELB	RMS acceleration	-0.135	0.135
Preferred-speed right turn	Left elbow	XPREFR	XPREFR_LELB_variability	PC2	20.100	XPREFR_saen_acc_LELB	Sample entropy, acceleration	-0.198	0.198
Preferred-speed right turn	Left elbow	XPREFR	XPREFR_LELB_variability	PC2	20.100	XPREFR_saen_gyr_LELB	Sample entropy, angular velocity	0.632	0.632
Preferred-speed right turn	Left thigh	XPREFR	XPREFR_LTHI_variability	PC1	77.587	XPREFR_rms_gyr_LTHI	RMS angular velocity	-0.446	0.446
Preferred-speed right turn	Left thigh	XPREFR	XPREFR_LTHI_variability	PC1	77.587	XPREFR_rms_acc_LTHI	RMS acceleration	-0.539	0.539
Preferred-speed right turn	Left thigh	XPREFR	XPREFR_LTHI_variability	PC1	77.587	XPREFR_saen_acc_LTHI	Sample entropy, acceleration	0.497	0.497
Preferred-speed right turn	Left thigh	XPREFR	XPREFR_LTHI_variability	PC1	77.587	XPREFR_saen_gyr_LTHI	Sample entropy, angular velocity	-0.513	0.513
Preferred-speed right turn	Left thigh	XPREFR	XPREFR_LTHI_variability	PC2	14.380	XPREFR_rms_gyr_LTHI	RMS angular velocity	-0.789	0.789
Preferred-speed right turn	Left thigh	XPREFR	XPREFR_LTHI_variability	PC2	14.380	XPREFR_rms_acc_LTHI	RMS acceleration	-0.139	0.139
Preferred-speed right turn	Left thigh	XPREFR	XPREFR_LTHI_variability	PC2	14.380	XPREFR_saen_acc_LTHI	Sample entropy, acceleration	-0.444	0.444
Preferred-speed right turn	Left thigh	XPREFR	XPREFR_LTHI_variability	PC2	14.380	XPREFR_saen_gyr_LTHI	Sample entropy, angular velocity	0.402	0.402
Preferred-speed right turn	Right elbow	XPREFR	XPREFR_RELB_variability	PC1	67.933	XPREFR_rms_gyr_RELB	RMS angular velocity	-0.479	0.479
Preferred-speed right turn	Right elbow	XPREFR	XPREFR_RELB_variability	PC1	67.933	XPREFR_rms_acc_RELB	RMS acceleration	-0.580	0.580
Preferred-speed right turn	Right elbow	XPREFR	XPREFR_RELB_variability	PC1	67.933	XPREFR_saen_acc_RELB	Sample entropy, acceleration	0.575	0.575
Preferred-speed right turn	Right elbow	XPREFR	XPREFR_RELB_variability	PC1	67.933	XPREFR_saen_gyr_RELB	Sample entropy, angular velocity	-0.323	0.323
Preferred-speed right turn	Right elbow	XPREFR	XPREFR_RELB_variability	PC2	25.355	XPREFR_rms_gyr_RELB	RMS angular velocity	-0.541	0.541
Preferred-speed right turn	Right elbow	XPREFR	XPREFR_RELB_variability	PC2	25.355	XPREFR_rms_acc_RELB	RMS acceleration	-0.146	0.146
Preferred-speed right turn	Right elbow	XPREFR	XPREFR_RELB_variability	PC2	25.355	XPREFR_saen_acc_RELB	Sample entropy, acceleration	-0.139	0.139
Preferred-speed right turn	Right elbow	XPREFR	XPREFR_RELB_variability	PC2	25.355	XPREFR_saen_gyr_RELB	Sample entropy, angular velocity	0.817	0.817
Preferred-speed right turn	Right thigh	XPREFR	XPREFR_RTHI_variability	PC1	73.603	XPREFR_rms_gyr_RTHI	RMS angular velocity	-0.453	0.453
Preferred-speed right turn	Right thigh	XPREFR	XPREFR_RTHI_variability	PC1	73.603	XPREFR_rms_acc_RTHI	RMS acceleration	-0.546	0.546
Preferred-speed right turn	Right thigh	XPREFR	XPREFR_RTHI_variability	PC1	73.603	XPREFR_saen_acc_RTHI	Sample entropy, acceleration	0.505	0.505
Preferred-speed right turn	Right thigh	XPREFR	XPREFR_RTHI_variability	PC1	73.603	XPREFR_saen_gyr_RTHI	Sample entropy, angular velocity	-0.492	0.492
Preferred-speed right turn	Right thigh	XPREFR	XPREFR_RTHI_variability	PC2	16.845	XPREFR_rms_gyr_RTHI	RMS angular velocity	-0.734	0.734
Preferred-speed right turn	Right thigh	XPREFR	XPREFR_RTHI_variability	PC2	16.845	XPREFR_rms_acc_RTHI	RMS acceleration	-0.211	0.211
Preferred-speed right turn	Right thigh	XPREFR	XPREFR_RTHI_variability	PC2	16.845	XPREFR_saen_acc_RTHI	Sample entropy, acceleration	-0.376	0.376
Preferred-speed right turn	Right thigh	XPREFR	XPREFR_RTHI_variability	PC2	16.845	XPREFR_saen_gyr_RTHI	Sample entropy, angular velocity	0.524	0.524

Notes: PC1 had its largest absolute loading on acceleration RMS in all 16 turning-condition × body-segment combinations. For PC2, the largest absolute loading was angular-velocity RMS in 12 combinations and angular-velocity sample entropy in four combinations. A loading sign indicates direction within the selected component orientation and should not be interpreted independently of the complete loading pattern.

Table S6. Selection frequencies of baseline limb-focused turning movement-dynamics features across fully nested leave-one-out cross-validation folds

fold	feature	selection_rank	partial_beta	screen_p	direction
0	XFASTR_RELB_movement_BL_PC2	1	2.602470369	0.188226966	1
0	XFASTL_RELB_movement_BL_PC2	2	1.935069296	0.236276118	1
0	XFASTL_LELB_movement_BL_PC2	3	-2.270155999	0.295770135	-1
0	XFASTR_LELB_movement_BL_PC1	4	-0.75550231	0.34331586	-1
0	XPREFR_RELB_movement_BL_PC2	5	1.67520142	0.405669937	1
1	XFASTL_LELB_movement_BL_PC2	1	-3.584858027	0.071209055	-1
1	XFASTR_LELB_movement_BL_PC1	2	-1.110961893	0.131542467	-1
1	XFASTL_LELB_movement_BL_PC1	3	-0.849373224	0.247440362	-1
1	XPREFL_LELB_movement_BL_PC1	4	-0.793205409	0.288698155	-1
1	XFASTR_LTHI_movement_BL_PC1	5	-0.769968655	0.28995358	-1
2	XFASTR_RELB_movement_BL_PC2	1	2.400984243	0.227418906	1
2	XFASTL_RELB_movement_BL_PC2	2	1.695774255	0.296893393	1
2	XFASTL_LELB_movement_BL_PC2	3	-2.138906852	0.328856722	-1
2	XFASTR_LELB_movement_BL_PC1	4	-0.727232965	0.362774925	-1
2	XPREFL_LELB_movement_BL_PC2	5	1.547933707	0.477766727	1
3	XFASTR_RELB_movement_BL_PC2	1	3.250832609	0.059127064	1
3	XPREFR_RELB_movement_BL_PC2	2	2.359855129	0.177558437	1
3	XFASTL_RELB_movement_BL_PC2	3	1.689164232	0.267202761	1
3	XPREFR_RTHI_movement_BL_PC2	4	2.531980859	0.269465562	1
3	XPREFL_RELB_movement_BL_PC2	5	1.617159604	0.278867446	1
4	XFASTR_RELB_movement_BL_PC2	1	2.588465577	0.208364741	1
4	XFASTL_RELB_movement_BL_PC2	2	1.7518093	0.290381092	1
4	XFASTL_LELB_movement_BL_PC2	3	-2.207453977	0.309176136	-1
4	XFASTR_LELB_movement_BL_PC1	4	-0.7453997	0.358110388	-1
4	XPREFL_LELB_movement_BL_PC2	5	1.693177259	0.442685068	1
5	XFASTR_RELB_movement_BL_PC2	1	2.820619815	0.140414813	1
5	XFASTL_LELB_movement_BL_PC2	2	-2.201504375	0.296906618	-1
5	XFASTL_RELB_movement_BL_PC2	3	1.638864121	0.305310326	1
5	XFASTR_LELB_movement_BL_PC1	4	-0.687900038	0.375419512	-1
5	XPREFR_RELB_movement_BL_PC2	5	1.459423002	0.449882169	1
6	XFASTR_RELB_movement_BL_PC2	1	2.523263971	0.192280627	1
6	XFASTL_RELB_movement_BL_PC2	2	1.954635143	0.223759443	1
6	XPREFL_LELB_movement_BL_PC2	3	2.520315614	0.263053925	1
6	XFASTR_LELB_movement_BL_PC1	4	-0.757703823	0.326977594	-1
6	XFASTL_LELB_movement_BL_PC2	5	-2.006272	0.351912394	-1
7	XFASTR_RELB_movement_BL_PC2	1	2.490972913	0.20826623	1
7	XFASTL_RELB_movement_BL_PC2	2	1.77922488	0.274139636	1
7	XFASTL_LELB_movement_BL_PC2	3	-2.352922021	0.284170184	-1
7	XFASTR_LELB_movement_BL_PC1	4	-0.771541758	0.335853195	-1
7	XPREFL_LELB_movement_BL_PC2	5	1.602752031	0.46398916	1
8	XFASTL_RELB_movement_BL_PC2	1	1.925347131	0.188033448	1
8	XFASTR_RELB_movement_BL_PC2	2	1.852380097	0.347592603	1
8	XFASTR_LELB_movement_BL_PC1	3	-0.541049577	0.465295527	-1
8	XFASTR_RTHI_movement_BL_PC1	4	-0.510655264	0.504308584	-1
8	XPREFR_RTHI_movement_BL_PC2	5	1.392808602	0.505144058	1
9	XFASTR_RELB_movement_BL_PC2	1	2.950611611	0.138636056	1
9	XFASTL_RELB_movement_BL_PC2	2	2.140993804	0.193074487	1
9	XFASTL_LELB_movement_BL_PC2	3	-2.306527612	0.283640747	-1
9	XFASTR_LELB_movement_BL_PC1	4	-0.778290116	0.325292628	-1
9	XPREFR_RELB_movement_BL_PC2	5	1.917547194	0.345147381	1
10	XFASTL_LELB_movement_BL_PC2	1	-3.069165459	0.12356363	-1
10	XFASTR_RELB_movement_BL_PC2	2	2.528033889	0.159632764	1
10	XFASTL_RELB_movement_BL_PC2	3	2.058990027	0.163218098	1
10	XFASTR_LTHI_movement_BL_PC2	4	-2.407015598	0.3167286	-1
10	XPREFR_RELB_movement_BL_PC2	5	1.58817387	0.376684383	1
11	XPREFL_LELB_movement_BL_PC2	1	2.695725494	0.233341422	1
11	XFASTR_RELB_movement_BL_PC2	2	2.077691226	0.351533223	1
11	XPREFR_LELB_movement_BL_PC2	3	2.268597091	0.359312718	1
11	XFASTR_LELB_movement_BL_PC1	4	-0.669131972	0.393798852	-1
11	XFASTL_LELB_movement_BL_PC2	5	-1.79379256	0.420208249	-1
12	XFASTL_LELB_movement_BL_PC2	1	-2.476251932	0.253730822	-1
12	XFASTR_RELB_movement_BL_PC2	2	2.262284536	0.271811566	1
12	XFASTL_RELB_movement_BL_PC2	3	1.556299955	0.338595789	1
12	XFASTR_LELB_movement_BL_PC1	4	-0.711040807	0.371402386	-1
12	XPREFL_LELB_movement_BL_PC2	5	1.610588619	0.455917989	1
13	XFASTR_RELB_movement_BL_PC2	1	3.003278133	0.111645263	1
13	XFASTL_RELB_movement_BL_PC2	2	2.38664438	0.128284335	1
13	XFASTL_LELB_movement_BL_PC2	3	-2.660784652	0.198702989	-1
13	XPREFR_RELB_movement_BL_PC2	4	1.978297315	0.301088903	1
13	XPREFL_RELB_movement_BL_PC2	5	1.478913156	0.379796807	1
14	XFASTL_LTHI_movement_BL_PC2	1	-2.956263694	0.032332393	-1

15	XFASTR_RELB_movement_BL_PC2	1	2.149312725	0.252998781	1
15	XFASTR_LTHI_movement_BL_PC2	2	-2.600948008	0.296936945	-1
15	XFASTR_RTHI_movement_BL_PC2	3	-2.262602027	0.327008359	-1
15	XFASTL_RELB_movement_BL_PC2	4	1.395601228	0.372820072	1
15	XFASTL_LELB_movement_BL_PC2	5	-1.650450484	0.419830146	-1
16	XPREFR_RTHI_movement_BL_PC2	1	2.767794371	0.169751743	1
16	XPREFL_RTHI_movement_BL_PC2	2	2.730169515	0.244495135	1
16	XFASTR_RELB_movement_BL_PC2	3	1.991357922	0.291320421	1
16	XPREFL_LELB_movement_BL_PC2	4	1.653762466	0.36390354	1
16	XPREFR_LELB_movement_BL_PC2	5	1.463499221	0.464985605	1
17	XFASTR_RELB_movement_BL_PC2	1	2.811186424	0.164260359	1
17	XFASTL_LELB_movement_BL_PC2	2	-2.771821733	0.223152347	-1
17	XFASTL_RELB_movement_BL_PC2	3	1.842053014	0.259098894	1
17	XFASTR_LELB_movement_BL_PC1	4	-0.736433034	0.356613066	-1
17	XPREFR_RELB_movement_BL_PC2	5	1.694198937	0.404555671	1
18	XFASTR_RELB_movement_BL_PC2	1	2.562380596	0.160052719	1
18	XFASTR_LELB_movement_BL_PC1	2	-0.915694073	0.215182665	-1
18	XPREFR_RELB_movement_BL_PC2	3	2.182509127	0.234756572	1
18	XFASTL_RELB_movement_BL_PC2	4	1.610227815	0.288729883	1
18	XFASTR_LTHI_movement_BL_PC2	5	-2.337794991	0.326822722	-1
19	XFASTR_RELB_movement_BL_PC2	1	2.360359047	0.235701309	1
19	XFASTL_RELB_movement_BL_PC2	2	1.692194027	0.294952579	1
19	XPREFL_LELB_movement_BL_PC2	3	2.37437631	0.299809953	1
19	XFASTL_LELB_movement_BL_PC2	4	-2.031780313	0.361127587	-1
19	XFASTR_LELB_movement_BL_PC1	5	-0.698118327	0.380842326	-1
20	XFASTR_RELB_movement_BL_PC2	1	2.509457795	0.206024431	1
20	XFASTL_RELB_movement_BL_PC2	2	1.867574903	0.260659518	1
20	XFASTL_LELB_movement_BL_PC2	3	-2.255964389	0.302219168	-1
20	XFASTR_LELB_movement_BL_PC1	4	-0.75137986	0.348274598	-1
20	XPREFR_RELB_movement_BL_PC2	5	1.458141331	0.46497414	1
21	XFASTR_RELB_movement_BL_PC2	1	2.461500981	0.213362454	1
21	XFASTL_RELB_movement_BL_PC2	2	1.758540698	0.27812176	1
21	XFASTL_LELB_movement_BL_PC2	3	-2.170002664	0.327273868	-1
21	XFASTR_LELB_movement_BL_PC1	4	-0.731624814	0.360655896	-1
21	XPREFL_LELB_movement_BL_PC2	5	1.673736619	0.444565137	1
22	XFASTR_RELB_movement_BL_PC2	1	2.375551919	0.222271833	1
22	XFASTL_RELB_movement_BL_PC2	2	1.732217942	0.276920477	1
22	XFASTR_LELB_movement_BL_PC1	3	-0.7517046	0.332363436	-1
22	XPREFL_LELB_movement_BL_PC2	4	1.913860572	0.388123643	1
22	XPREFR_RELB_movement_BL_PC2	5	1.396787416	0.472548654	1

Notes: Selection frequency indicates the proportion of the 23 LOOCV training folds in which a task–segment–PC feature was retained. It does not indicate repeated statistical significance or effect magnitude. When no feature met the training-fold screening criterion of $p < 0.05$, the five lowest-p-value features were retained to construct the fold-specific signature. Component signs are arbitrary; therefore, physiological direction should be interpreted from the source-feature loading pattern rather than from the sign of the PC score alone.
